# Translocation of TMEM175 Lysosomal Potassium Channel to the Plasma Membrane by Dynasore Compounds

**DOI:** 10.3390/ijms221910515

**Published:** 2021-09-29

**Authors:** Enikő Pergel, Irén Veres, Gergely Imre Csigi, Gábor Czirják

**Affiliations:** Department of Physiology, Semmelweis University, 1094 Budapest, Hungary; pergel.eniko@med.semmelweis-univ.hu (E.P.); veres.iren@med.semmelweis-univ.hu (I.V.); csigiger@gmail.com (G.I.C.)

**Keywords:** dynasore, electrophysiology, endocytosis, endosome, lysosome, plasma membrane, potassium channel, SC79, *Xenopus laevis*

## Abstract

TMEM175 (transmembrane protein 175) coding sequence variants are associated with increased risk of Parkinson’s disease. TMEM175 is the ubiquitous lysosomal K^+^ channel regulated by growth factor receptor signaling and direct interaction with protein kinase B (PKB/Akt). In the present study, we show that the expression of mouse TMEM175 results in very small K^+^ currents through the plasma membrane in *Xenopus laevis* oocytes, in good accordance with the previously reported intracellular localization of the channel. However, the application of the dynamin inhibitor compounds, dynasore or dyngo-4a, substantially increased TMEM175 currents measured by the two-electrode voltage clamp method. TMEM175 was more permeable to cesium than potassium ions, voltage-dependently blocked by 4-aminopyridine (4-AP), and slightly inhibited by extracellular acidification. Immunocytochemistry experiments indicated that dyngo-4a increased the amount of epitope-tagged TMEM175 channel on the cell surface. The coexpression of dominant-negative dynamin, and the inhibition of clathrin- or caveolin-dependent endocytosis increased TMEM175 current much less than dynasore. Therefore, dynamin-independent pharmacological effects of dynasore may also contribute to the action on the channel. TMEM175 current rapidly decays after the withdrawal of dynasore, raising the possibility that an efficient internalization mechanism removes the channel from the plasma membrane. Dyngo-4a induced about 20-fold larger TMEM175 currents than the PKB activator SC79, or the coexpression of a constitutively active mutant PKB with the channel. In contrast, the allosteric PKB inhibitor MK2206 diminished the TMEM175 current in the presence of dyngo-4a. These data suggest that, in addition to the lysosomes, PKB-dependent regulation also influences TMEM175 current in the plasma membrane.

## 1. Introduction

TMEM175 coding sequence variants are associated with the development of Parkinson’s disease [1,2,3,4,5,6,7,8,9]. TMEM175 has recently been identified as the ubiquitous K^+^ channel of endosomal and lysosomal membranes [10]. Despite its importance, only a few studies have been reported about the electrophysiological properties of TMEM175 [10,11,12,13,14], in part because of the problematic accessibility of the native channel in the intracellular organelles. TMEM175 has an unusual ion selectivity profile, as determined in patch clamp measurements of endosomes enlarged by Rab5-Q79L transfection. In these experimentally manipulated organelles, TMEM175 was found to be more permeable to Cs^+^ than K^+^, in contrast to several other K^+^ channel types of the plasma membrane, which are generally blocked by Cs^+^. TMEM175 is inhibited by 4-aminopyridine (4-AP), an inhibitor of voltage-gated potassium channels, however, 4-AP is also effective on the maintained K^+^ current of TMEM175 at negative membrane potential values [10]. Two groups also reported K^+^ currents of approximately 200 pA, measured in HEK293 cells overexpressing mammalian TMEM175, by the whole cell patch clamp method [11,13]. These TMEM175 currents exceeded the endogenous K^+^ currents of non-expressing cells, and allowed the examination of channel function, however, did not reach the nA range, which is characteristic for the expression of other K^+^ channel types normally targeted to the plasma membrane.

In the present study, we introduce a pharmacological approach to induce sufficient TMEM175 current for conventional electrophysiology in a widely used expression system, in the plasma membrane of *Xenopus laevis* oocytes. Ion selectivity, pharmacological sensitivity, and regulation by intracellular signaling are maintained in the case of surface expression, suggesting that the basic properties of the channel in the plasma membrane are similar to those of TMEM175 at the physiological location. Measuring currents one or two orders of magnitude larger than before may expedite the investigation of TMEM175, by revealing fine details, and reducing the relative contribution of endogenous cellular conductance to the recording.

TMEM175 currents have not yet been investigated by the two-electrode voltage clamp technique in *Xenopus* oocytes (to our knowledge). In our experiments, the heterologous expression of TMEM175, the microinjection of high amounts (12 ng) of mouse TMEM175 cRNA resulted in small (<1 µA) currents. In most cell preparations, only 0.2–0.4 µA was measured. This low level of functional expression interfered with the reliable discrimination of TMEM175 current from the endogenous conductance of the oocyte membrane. Considering the small, but detectable current expression, we investigated whether the internalization of TMEM175 contributes to the limited abundance of the channel in the plasma membrane. We tested the effect of different inhibitors of endocytosis, and among them, identified the dynasore compounds as highly efficient agents inducing robust TMEM175 currents in the plasma membrane.

## 2. Results

### 2.1. Dynasore and Dyngo-4a Induce TMEM175 Current in the Plasma Membrane

**Figure 1:**
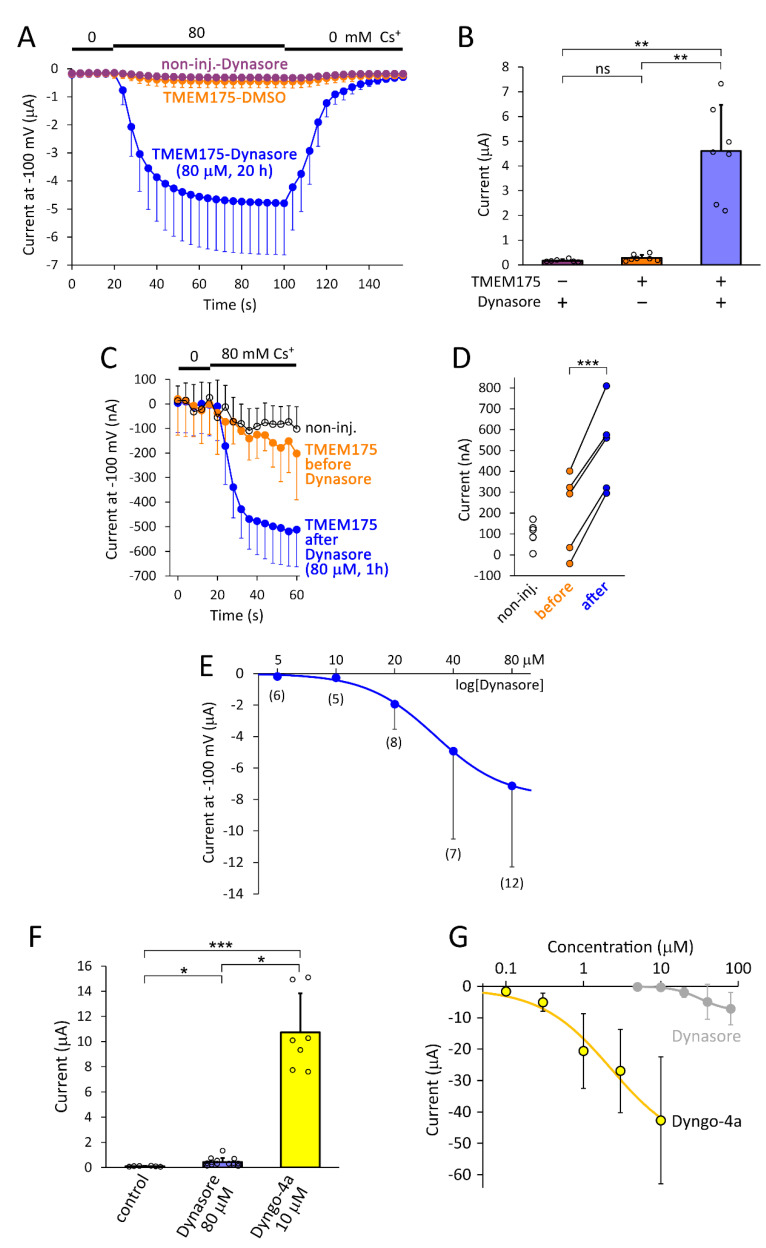
**Dynasore and dyngo-4a increase TMEM175 current in the plasma membrane.** (**A**). *Xenopus* oocytes expressing mouse TMEM175 and non-injected control cells (*non-inj.*) were treated with dynasore (80 μM, 20 h) or vehicle (DMSO), as indicated on the *graph*. The currents were measured by two-electrode voltage clamp at −100 mV, when the extracellular NMDG solution was changed to 80 mM [Cs^+^] and back, as indicated above the curves (*n* = 6 or 7 cells, in each group). (**B**). Statistical evaluation of the effects of TMEM175 expression and dynasore on the Cs^+^ currents (as indicated below the columns, using the data plotted in *panel A*). The oocytes derived from the same cell preparation. ** *p* < 0.005 (Welch’s ANOVA, Dunnett T3 post hoc test) (**C**). The average cesium currents in the oocytes expressing TMEM175, before (*orange*) and after the short dynasore treatment (80 μM, 1 h, *blue*, *n* = 5, the cells were measured twice). As a reference, the currents of non-injected cells without dynasore treatment are also shown (*black*, *n* = 5). (**D**). Statistical evaluation of the data plotted in panel C. The increase of TMEM175 current in response to dynasore (1 h) is shown for each cell. *** *p* < 0.001 (paired *t*-test) (**E**). Dose-response relationship between dynasore concentration (5, 10, 20, 40, 80 µM) and TMEM175 Cs^+^ current. Dynasore was applied overnight (for 20 h). The numbers in the brackets indicate sample size. The cells derived from two oocyte preparations. (**F**). Three groups of oocytes expressing TMEM175 were incubated in the presence of *Dynasore* (80 µM, *n* = 14) or *Dyngo-4a* (10 µM, *n* = 7) for 20 h, or in control solution (*control*, *n* = 6). The oocytes derived from the same cell preparation. TMEM175 current was measured in 80 mM EC K^+^ at −100 mV. * *p* < 0.05, *** *p* < 10^−4^, (Kruskal–Wallis ANOVA) (**G**). Dose-response relationship between Dyngo-4a concentration (0.1, 0.3, 1, 3, 10 µM) and TMEM175 K^+^ current was determined in another oocyte preparation (*n* = 5, for each concentration, applied for 20 h). As an illustration, the dynasore dose-response curve from panel E is also included (*grey curve*), although it has been measured in different cell preparations and with Cs^+^ instead of K^+^. (Error bars indicate standard deviation (S.D.), ns: not significant).

In a preparation of *Xenopus laevis* oocytes expressing mouse TMEM175 channel, the average cesium current was 0.29 ± 0.13 µA in the plasma membrane, measured by the two-electrode voltage clamp technique in 80 mM extracellular (EC) [Cs^+^], at −100 mV (*n* = 7, Figure 1A,B). In sharp contrast, in response to the overnight application of dynasore (80 µM, 20 h), the cesium current increased to 4.6 ± 1.9 µA (*n* = 7, *p* < 0.005). The same dynasore treatment did not result in similar Cs^+^ conductance in the non-injected control oocytes (0.18 ± 0.06 µA, *n* = 6, *p* < 0.005, compared to the currents induced by dynasore in the cells expressing TMEM175). Dynasore increased the average TMEM175 current approximately 40-fold, if the baseline of non-injected cells is subtracted in the calculation; indeed, even without this correction, the increase is substantial, at about 16-fold. In this experiment, the currents were measured in 80 mM EC [Cs^+^] in order to better discriminate TMEM175 from the endogenous K^+^ channels, and at −100 mV, to suppress the depolarization-activated current components of the oocytes.

The above chronic treatment of the cells with dynasore may exert diverse effects, possibly including the alteration of gene expression. In order to exclude the contribution of chronic changes, short application of dynasore was also tested. We measured the basal cesium current of TMEM175-expressing oocytes, incubated them in 80 µM dynasore for an hour, and measured the current again in the same cell (Figure 1C,D). TMEM175 current was increased by the short dynasore treatment in all cells, on average by 310 ± 64 nA (*n* = 5, *p* < 0.001, Figure 1D). Apparently, long-term effects are not necessary; dynasore increases TMEM175 current in an hour.

Dynasore is a widely used inhibitor of dynamin, the half maximal inhibitory concentration (IC_50_) was reported to be about 15 µM [15]. We have determined the dose-response relationship between dynasore concentration and TMEM175 current (Figure 1E). The maximum TMEM175 current (saturation) at high inhibitor concentration could not be reached, because of the toxicity of the compound on the oocytes. Therefore, the Hill equation fit only provides a low estimate of the half maximal effective concentration (EC_50_); the EC_50_ is not less than approximately 30 µM. This EC_50_ is close to the reported IC_50_ for dynamin inhibition, and it is consistent with the hypothesis that the inhibition of dynamin contributes to the functional expression of TMEM175 current in the plasma membrane.

Dynasore significantly increased TMEM175 current in all experiments. However, the effect of the dynamin inhibitor was highly variable among the different oocyte preparations (see supplementary Figure S1), indicating that it is essential to compare groups of oocytes from the same animal in further experiments. Dyngo-4a, the hydroxylated derivative of dynasore, inhibits dynamin with a higher potency than the parent compound; IC_50_ for the in vitro inhibition of dynamin-2 was reported to be 2.3 µM [16]. We compared the effects of dyngo-4a and dynasore on TMEM175 current in the plasma membrane of oocytes from the same preparation (Figure 1F). In this cell preparation, dynasore (80 µM) exerted a small but significant effect on TMEM175 current. However, dyngo-4a (10 µM) increased TMEM175 K^+^ currents to much higher values (10.7 ± 3.1 µA, *n* = 7, *p* < 10^−4^ compared to non-injected, *p* < 0.02 compared to the dynasore-treated cells). In our experience, dyngo-4a proved to be an even better tool to potently increase TMEM175 current in the plasma membrane than dynasore, although the effect of dyngo-4a was also characterized by biological variation among the different cell preparations (supplementary Figure S1).

The dose-response relationship between dyngo-4a and TMEM175 K^+^ current was determined in the oocytes from another preparation (Figure 1G). The maximum TMEM175 current at high inhibitor concentration could not be reached, because of the limited solubility of dyngo-4a. Thus, the Hill curve fit provided a lower estimate that the EC_50_ is not less than 2.3 µM (which is exactly the same as the reported IC_50_ for the inhibition of dynamin). The potency of dyngo-4a for the induction of TMEM175 current follows the potency for dynamin inhibition, which is increased by the chemical modification of dynasore.

### 2.2. TMEM175 Approximates the Ideal Background Potassium Channel

**Figure 2:**
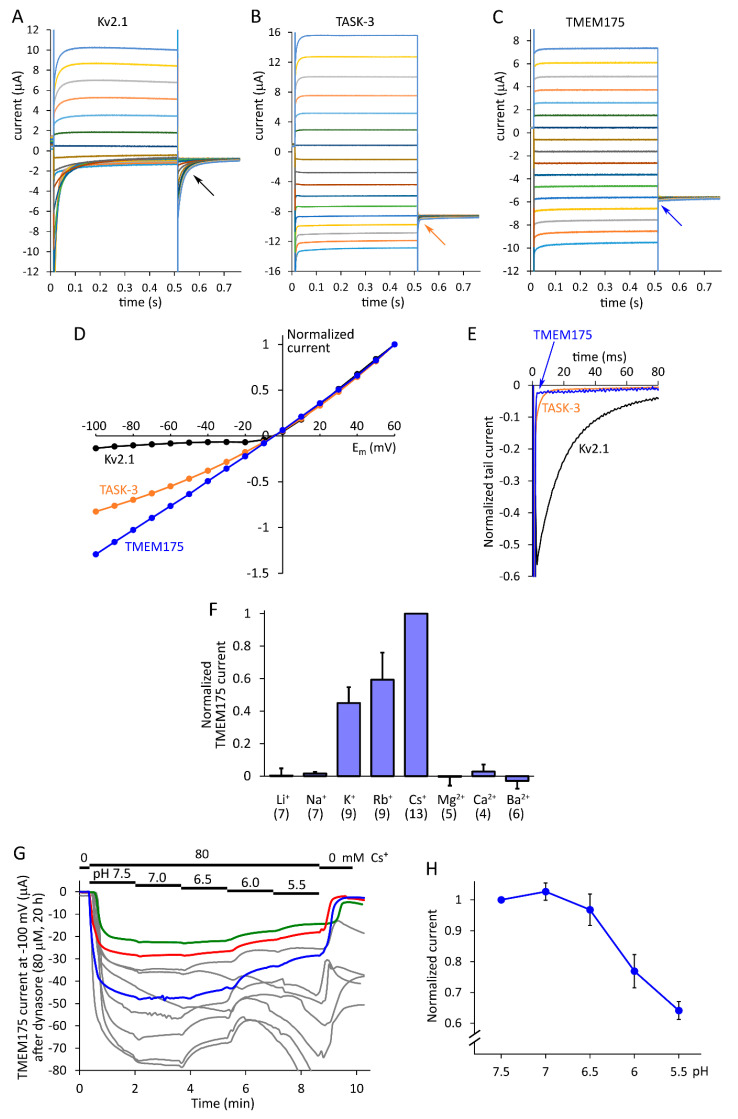
**TMEM175 was compared to K_V_2.1 and TASK-3 under identical conditions, and TMEM175 ion selectivity and pH-sensitivity were determined in the plasma membrane**. (**A**–**C**). The currents of three representative oocytes expressing rat K_V_2.1, mouse TASK-3, or mouse TMEM175 were measured in 80 mM EC K^+^, during 500 ms-long voltage steps from −100 to +60 mV in 10 mV increments, from a holding potential of 0 mV. This was followed by a voltage step to −60 mV, in order to record the deactivation kinetics (tail currents, as indicated by the *oblique arrows*). TMEM175 current was induced by the application of dyngo-4a (10 µM, 20 h). Representative recordings with similar current amplitudes and reversal potential (close to −5 mV in 80 mM EC K^+^) were selected. The non-specific leak (in 2 mM EC K^+^ at −100 mV) was negligible in these cells. (**D**). Current-voltage (I–V) relationships of the three channel types measured at 0.5 s in panels (A–C). The currents were normalized to the maximum value at +60 mV. (For further data, see supplementary Figure S2.) (**E**). Normalized tail currents at −60 mV, following the +60 mV voltage steps from the three (**A**–**C**) recordings. The current at the end of the voltage step to −60 mV was taken as the baseline, and the tail current amplitudes were normalized to the value measured at the end of the voltage step to +60 mV. (**F**). The ion selectivity profile of mouse TMEM175 was determined in the plasma membrane of *Xenopus* oocytes treated with dynasore (80 µM, 20 h). The currents were measured at −100 mV with two-electrode voltage clamp in extracellular solutions containing 80 mM of the cations indicated below the graph (the divalent cation solutions were hyperosmotic). The non-specific leak current measured in NMDG was subtracted, and the data were normalized to the Cs^+^ current in each cell. The numbers in the brackets indicate sample size. (**G**). The sensitivity of TMEM175 to acidification was measured in *Xenopus* oocytes. Considering the standard topology of vesicular insertion into the plasma membrane, the lysosomal luminal side of TMEM175 is exposed to the EC space. The pH was gradually decreased in the presence of 80 mM Cs^+^, as indicated above the recordings. Each curve represents the measurement of a cell. In the majority of recordings (*grey curves*), the acidification activated an endogenous conductance of the oocyte. Thus we could only test the effect of acidification to pH 5.5, although lysosomal pH may be as low as 4.5. The three recordings (*green, red* and *blue*), which returned to baseline at the end of the measurement in the NMDG solution (*0 mM Cs^+^*), were selected for further evaluation, because these were not affected by the pH-sensitive endogenous conductance. Dynasore (80 µM, 20 h) induced robust TMEM175 currents in this cell preparation. (**H**). Normalized TMEM175 current amplitudes at different pH values. Data points were calculated from the recordings in *panel G* (*n* = 3). Note the discontinuous vertical axis.

We compared TMEM175 to K_V_2.1 voltage-gated and TASK-3 K_2P_ background K^+^ currents in the plasma membrane, in the same oocyte preparation under identical conditions (Figure 2). This unbiased comparison of the different currents could not be possible without the application of dyngo-4a on the cells expressing TMEM175. K_V_2.1, as a member of the voltage-gated potassium channel family, showed considerably higher voltage-dependent activation after the depolarizing voltage steps than the background (leak) K^+^ channel TASK-3. However, the voltage-dependently increasing current component was absent in the TMEM175-expressing cell, even in the case of strong depolarization, after the beginning of the voltage step to +60 mV (compare the *upper light blue curves* in Figure 2A–C). In the TMEM175 recording, the small activating component may be in the range of endogenous voltage-dependent currents of the oocyte, thus TMEM175 is not activated in a voltage-dependent manner, i.e., it is less activated than the canonical background K^+^ channel TASK-3.

Depolarization increases TASK-3 steady-state current more at positive than at negative membrane potentials (distances between the *horizontal lines* are not equal in Figure 2B), in accordance with the ion-flux coupled, voltage-dependent gating mechanism of K_2P_ channels [17]. However, this phenomenon cannot be observed in the TMEM175 recording, the current curves are almost equally spaced (Figure 2C), and the current-voltage relationship is nearly linear (Figure 2D), as it is expected for an ideal background potassium channel in symmetrical K^+^ concentrations, on the basis of the Goldman–Hodgkin–Katz theory [18].

Slow deactivation of Kv2.1 produced large tail currents during the voltage steps to −60 mV after the depolarizing prepulses (*black arrow* in Figure 2A, and *black curve* in Figure 2E). The tail currents of Kv2.1 are more prominent than those of TASK-3, because of the limited voltage-dependence and rapid deactivation of K_2P_ channels (*orange arrow* in Figure 2B, and *orange curve* in Figure 2E). However, the tail currents of TMEM175 could not be detected (*blue arrow* in Figure 2C), the exponentially decaying component is not present (Figure 2E), in good accordance with the lack of voltage-dependent activation. In summary, with respect to its current kinetics, TMEM175 more completely fulfils the criteria for an ideal background potassium channel than TASK-3.

### 2.3. Ion Selectivity, pH-Sensitivity and Inhibition by 4-Aminopyridine

TMEM175 expressed in the plasma membrane is permeable to Cs^+^ > Rb^+^ > K^+^, and practically impermeable to Li^+^, Na^+^ and divalent cations (Figure 2F). The potassium current conducted by TMEM175 channel was about 45% of the Cs^+^ current at −100 mV, in 80 mM EC ion concentrations. The inward current in K^+^ was about 29-times larger than in Na^+^, in good accordance with the remarkable K^+^ selectivity of the channel. The peculiar ion selectivity profile verifies TMEM175 expression in the plasma membrane. The ion selectivity is not apparently changed by the lipid composition of the plasma membrane, which is different from the lysosomes [10].

The gradual acidification during endosome maturation and low lysosomal pH have paramount importance in the normal function of these organelles [19,20]. Genetic dysfunction of TMEM175 has been reported to interfere with lysosomal pH stabilization [10,21,22], however, to our knowledge, the direct effect of pH on TMEM175 has not yet been investigated. We found that EC acidification from 7.5 to 5.5 inhibited TMEM175 by 36 ± 3% (*n* = 3, Figure 2G,H). Thus, TMEM175 is inhibited by the acidification, similarly to the majority of other K^+^ channel types, however, it is inhibited less efficiently than the canonical pH-sensor K_2P_ channels of the TASK family [23]. Regarding the drastic acidification in the endosomal/lysosomal system by 2–3 pH units, the pH-sensitivity of TMEM175 may have biological significance.

**Figure 3:**
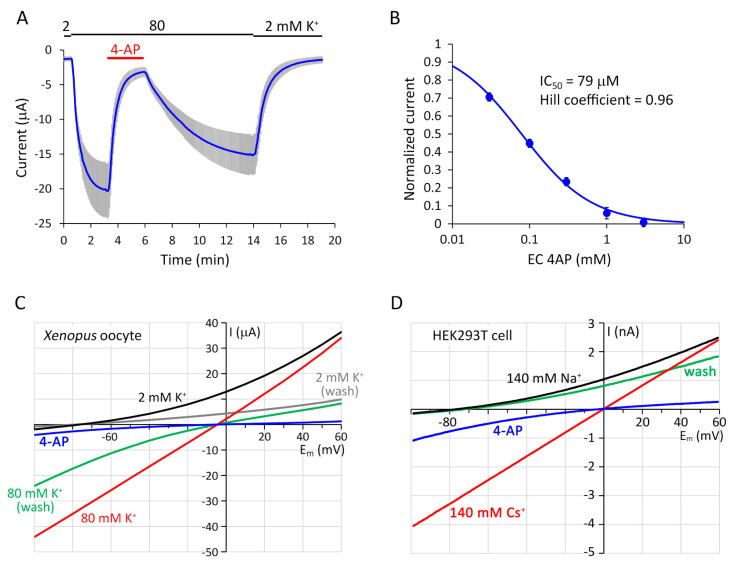
**Voltage-dependent inhibition of TMEM175 by 4-aminopyridine (4-AP).** (**A**). TMEM175 current is robustly inhibited by extracellular (EC) 4-aminopyridine at −100 mV (*4-AP*, 1 mM, *red bar*). Average TMEM175 currents are plotted (*n* = 5). The EC K^+^ concentration was changed from 2 to 80 mM and back as indicated *above the graph*. (**B**). Dose-response relationship between EC 4-AP concentration and TMEM175 current. The current was measured at −100 mV in 80 mM EC K^+^, in the presence of gradually increasing concentrations of 4-AP (0.03, 0.1, 0.3, 1, 3 mM; *n* = 5 cells treated overnight with 10 µM dyngo-4a). (**C**). Current-voltage (I–V) relationships of mouse TMEM175 in different EC solutions, in an oocyte treated with 10 µM dyngo-4a for 20 h. The solutions were applied in the following order: 2 mM K^+^ (*black curve*), 80 mM K^+^ (*red curve*), 1 mM 4-AP in the presence of 80 mM K^+^ (*blue curve*), 80 mM K^+^ after 300 s washout of 4-AP (*wash, green curve*), and finally 2 mM K^+^ again (*wash, grey curve*). The currents were measured at the end of 500-ms voltage steps from −100 mV to +60 mV in 10 mV increments, and the data points were interpolated with line segments. Representative of three similar recordings. (**D**). Current-voltage (I–V) relationships of mouse TMEM175 expressed in a HEK293T cell, treated overnight with 80 μM dynasore. The currents were measured by voltage ramps from −100 to +60 mV for 800 ms, in different EC solutions, containing: 140 mM Na^+^ (*black*), 140 mM Cs^+^ (*red*), 1 mM 4-aminopyridine in the presence of 140 mM Cs^+^ (*4-AP, blue*), and 140 mM Na^+^ again after the washout of 4-AP (*wash, green*). The pipette solution contained 140 mM Cs^+^. Representative of four similar recordings.

The application of 4-aminopyridine (4-AP, 1 mM) in the EC space resulted in substantial inhibition of TMEM175 current at −100 mV in 80 mM EC K^+^ (90.0 ± 1.8% inhibition, *n* = 5, Figure 3A). The IC_50_ 78.7 ± 0.5 µM (*n* = 5, Figure 3B) is close to the previously reported 35 µM value, which was measured in 150 mM K^+^ at +100 mV by patch clamping endosomes dilated with Rab5-Q79L overexpression, i.e., with opposite current direction and 4-AP applied on the other (cytoplasmic) side of the membrane [10]. We observed slightly voltage-dependent inhibition of mouse TMEM175 by 4-AP (Figure 3C,D), in contrast to the previously reported voltage-independent inhibition of human TMEM175 in HEK293T cells [13]. TMEM175 current was more profoundly inhibited by 4-AP at +60 mV than at −60 mV, suggesting that the positively charged inhibitor binds into the pore from the cytoplasmic direction. The slow recovery of the current from the inhibition after the removal of extracellular 4-AP (Figure 3A) may also correspond to the accumulation of the protonated form of the membrane-permeable inhibitor in the cytoplasm, as in the case of the voltage-gated K_V_1 channels [24,25].

### 2.4. The Amount of TMEM175 Protein Is Increased in the Plasma Membrane by Dyngo-4a

The dynasore compounds may exert their effect on TMEM175 current by increasing the number of channels in the plasma membrane or by the stimulation of channel activity. It is difficult to precisely distinguish between these two mechanisms. In order to gain insight into the changes of TMEM175 surface expression by dyngo-4a, we estimated the amount of channel protein in the plasma membrane by two different methods.

**Figure 4:**
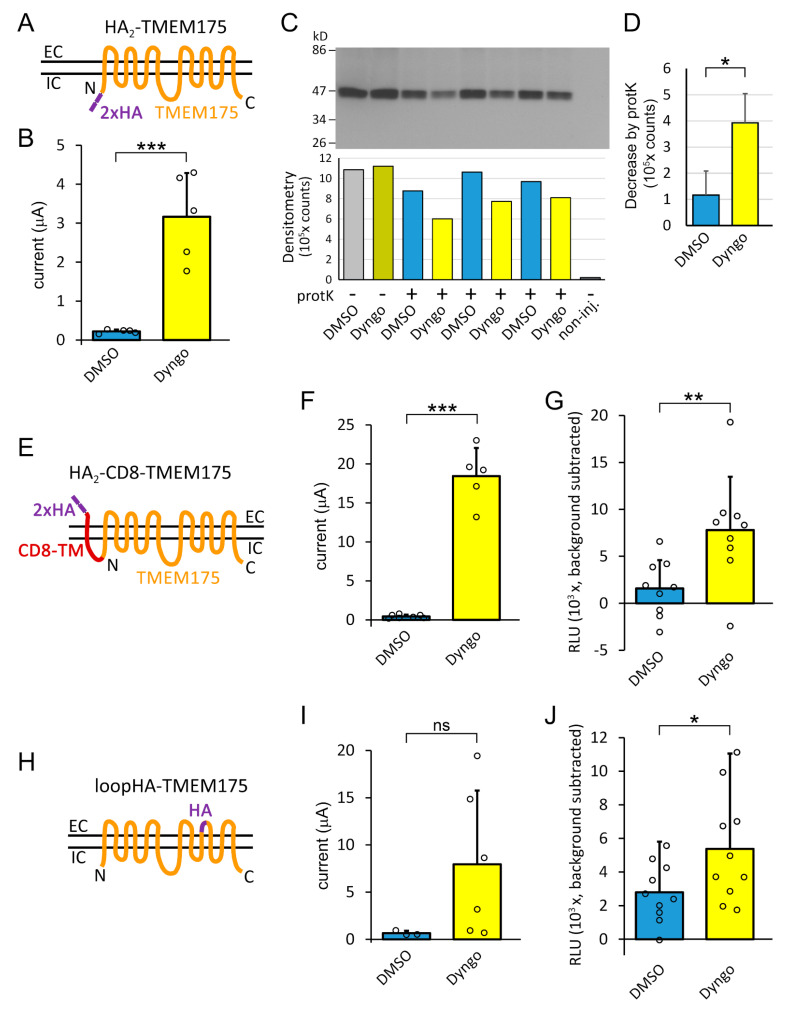
**Dyngo-4a increases the amount of TMEM175 protein in the plasma membrane.** (**A**). Schematic transmembrane topology of the HA_2_-TMEM175 construct with the intracellular, *N*-terminal double influenza haemagglutinin (HA) tag. (**B**). The currents of the construct introduced in *panel A* are increased by the treatment of the oocytes with dyngo-4a (10 μM, 20 h, *** *p* < 0.005 compared to DMSO, Mann–Whitney U-test). The currents were measured in 80 mM K^+^ at −100 mV. (**C**). Anti-HA immunoblot of the crude plasma membrane preparations from the oocytes expressing HA_2_-TMEM175 are shown. Eight groups of cells (*n* = 19 oocytes in each group) were treated with dyngo-4a (10 μM, *Dyngo*) or vehicle (*DMSO*), and in six groups, the proteins on the cell surface were digested with proteinase K (*protK*), as indicated below the *graph*. The proteolytic fragments are not visible on the immunoblot. No signal was detected in the control non-injected cells (*non-inj.*, *n* = 19). Densitometry analysis of the bands is shown below the immunoblot. Note the lower intensity of bands in the Dyngo than in the DMSO groups. (**D**). Statistical analysis of the decrease of the anti-HA signals by proteinase K in the *Dyngo* and *DMSO* groups. * *p* < 0.05, (Student’s *t*-test, unpaired, homoscedastic) (**E**). TMEM175 structure was extended *N*-terminally with the single transmembrane segment of human CD8 protein, and an extracellular double-HA-tag was appended at the N-terminus. (**F**). The currents of the HA_2_-CD8-TMEM175 construct introduced in panel E are increased by the treatment of the oocytes with dyngo-4a (10 μM, 20 h, *** *p* < 0.005 compared to DMSO, Mann–Whitney U-test). The currents were measured in 80 mM K^+^ at −100 mV. (**G**). Luminometry data (given in relative light units, RLU) of the oocytes expressing HA_2_-CD8-TMEM175, and treated with dyngo-4a (10 μM, *Dyngo*) or vehicle (*DMSO*), as indicated below the *graph*. The signals were obtained by anti-HA indirect immunocytochemistry of fixed cells, followed by on cell surface horseradish peroxidase enhanced chemoluminescence (HRP-ECL) reaction. The background was determined by the identical reaction of non-injected oocytes. ** *p* < 0.02, (Student’s *t*-test, unpaired, homoscedastic) (**H**). TMEM175 was extracellularly HA-tagged by replacing a sequence of amino acids in the fifth EC loop with the HA epitope. (**I**). The currents of the loopHA-TMEM175 construct introduced in *panel H* shows the tendency to be increased by the treatment of the oocytes with dyngo-4a (10 μM, 20 h, *p* = 0.07 compared to DMSO, Mann–Whitney U-test). The currents were measured in 80 mM K^+^ at −100 mV. (**J**). Luminometry data with loopHA-TMEM175 in a similar experiment, as shown in *panel G*. * *p* < 0.05, (Student’s *t*-test, unpaired, heteroscedastic).

In the first approach, TMEM175 channels in the plasma membrane were selectively targeted by extracellular digestion of intact *Xenopus* oocytes with proteinase K. This protease non-specifically cleaves the proteins in the plasma membrane, but it does not have access to TMEM175 located in the membranes of intracellular organelles. We compared eight groups of oocytes, each containing 19 cells, expressing the HA_2_-TMEM175 construct tagged at the *N*-terminus with double influenza hemagglutinin (HA) epitope (Figure 4A). We verified that dyngo-4a increased the current of this HA-tagged construct in the plasma membrane (Figure 4B). The groups of oocytes were treated with dyngo-4a (10 μM) or the vehicle DMSO for 20 h, were or were not digested with proteinase K for 45 min (as indicated in Figure 4C), and subsequently crude plasma membrane fractions were isolated after homogenization in the presence of protease inhibitors. These fractions were analyzed by anti-HA immunoblot and densitometry (Figure 4C).

After the treatment with dyngo-4a, a higher amount of TMEM175 channels was accessible to proteinase K than after the application of the vehicle DMSO (Figure 4C). Accordingly, the anti-HA signal intensity in the *Dyngo* groups was more profoundly decreased by the digestion of the surface of the oocytes than in the control *DMSO* groups. The differences of the *yellow* 4th, 6th, and 8th columns from the 2nd column are larger than the differences of the *blue* 3rd, 5th and 7th columns from the first *grey* one in Figure 4C. The reductions of signal intensity by proteinase K are significantly different between the *Dyngo* and *DMSO* groups (*n* = 3 groups of oocytes for both dyngo-4a and DMSO, *p* < 0.05, Figure 4D). The more profound decrease of the signal in the *Dyngo* group than in the *DMSO* group suggests that the number of TMEM175 channels was increased on the cell surface by the treatment with dyngo-4a.

Dyngo-4a also reduced the amount of TMEM175 proteins not cleaved by the protease (compare the 4th, 6th, and 8th vs. the 3rd, 5th and 7th columns in Figure 4C, *p* < 0.05, Student’s *t*-test). This fraction of TMEM175 may correspond to the channels of intracellular compartments, co-purifying with the plasma membrane preparation. The translocation of TMEM175 to the plasma membrane may contribute to the decreased amount of channels in the intracellular organelles, although unequivocal interpretation of the complex changes of the protease-resistant TMEM175 fraction are not possible in this experimental design. In principle, the digestion of TMEM175 in the first EC loop by proteinase K results in a small proteolytic fragment containing the double-HA-tag, with a calculated molecular weight of 8.8 kD, however, this small fragment was not detected on the immunoblot.

In order to confirm the increase of channel number in the plasma membrane by dyngo-4a, we have also taken another approach. We applied two strategies to design functional TMEM175 constructs with extracellular HA-tags. In the HA_2_-CD8-TMEM175 construct, a fragment of human CD8 protein containing a single transmembrane segment was connected to the *N*-terminus of TMEM175, and also appended with an extracellular double HA-tag at the *N*-terminus of the CD8 fragment (Figure 4E). In the other loopHA-TMEM175 construct, a short region of the fifth extracellular loop of TMEM175 was replaced with the HA-tag amino acid sequence (Figure 4H). The CD8-based construct was clearly functional, and responsive to dyngo-4a (Figure 4F). The internally tagged construct also showed the tendency for increased K^+^ current in response to the application of dyngo-4a (*p* = 0.07, Figure 4I).

The surface expression of the extracellularly HA-tagged TMEM175 constructs was examined by indirect anti-HA immunocytochemistry and on-cell horseradish peroxidase-based enhanced chemiluminescence (HRP-ECL) reaction of fixed *Xenopus* oocytes, followed by luminometry. The plasma membrane was not permeabilized in these experiments. The treatment of the cells with dyngo-4a (10 μM, 20 h) significantly increased the luminometry signal of both constructs, compared to the vehicle DMSO (Figure 4G,J). The abundant anti-HA immunoreactivity on the cell surface after the application of dyngo-4a indicates that the number of channels was increased in the plasma membrane.

The results from both biochemical approaches support the conclusion that the amount of TMEM175 protein is increased by dyngo-4a in the plasma membrane. Nevertheless, both approaches provided semiquantitative data, therefore we cannot exclude the possibility that the channel activity increased by dyngo-4a also contributes to the robust current expression in the plasma membrane, in addition to the enhanced surface expression of TMEM175 channels.

**Figure 5:**
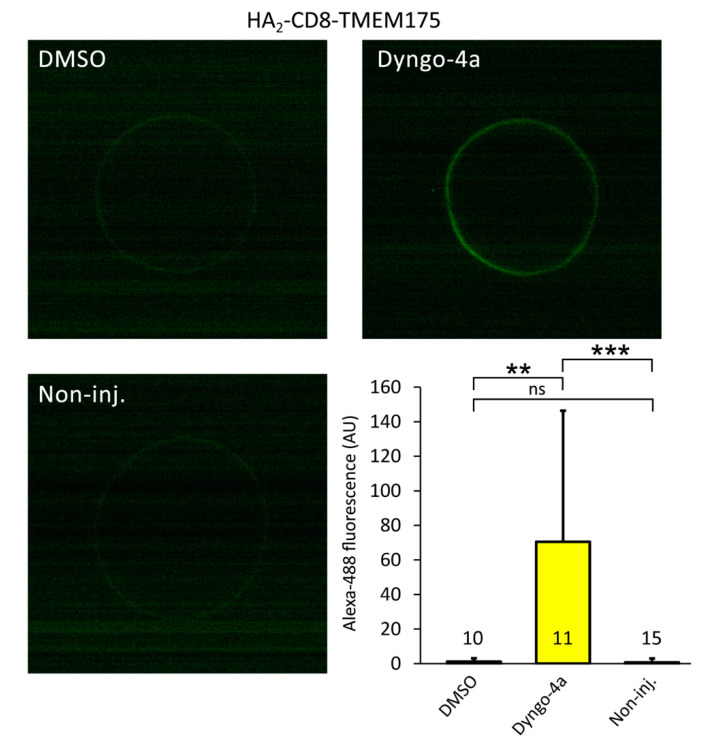
**Confocal microscopy indicates that TMEM175 surface expression is increased after the application of dyngo-4a.** The *upper* two images show the representative optical sections of *Xenopus* oocytes expressing the HA_2_-CD8-TMEM175 construct, and fixed after the application of *Dyngo-4a* (10 μM, 20 h) or *DMSO*, as indicated on the panels. The extracellular HA-tags were detected by the binding of a mouse anti-HA primary antibody, followed by an anti-mouse secondary antibody conjugated with Alexa Fluor 488. The *left lower panel* (*Non-inj.*) shows the background immunofluorescence of a representative non-injected control oocyte after identical immunostaining. The surface immunofluorescence was significantly more intense after the application of dyngo-4a on the oocytes expressing HA_2_-CD8-TMEM175 than after the treatment with DMSO, or that of the non-injected cells, as indicated on the *column graph*. The numbers above (or in) the *columns* indicate the number of measured oocytes. For the whole image set, see supplementary Figure S4. ** *p* < 0.01, *** *p* < 10^−4^ (multiple comparisons after Kruskal–Wallis ANOVA), ns: not significant.

We have also visualized the increased surface expression of TMEM175 by confocal microscopy. The detection of EGFP-TMEM175, a fusion construct containing enhanced green fluorescent protein connected to the *N*-terminus of the channel subunit, was not an adequate tool to estimate surface expression (supplementary Figure S3). Therefore, we used the TMEM175 constructs with the extracellular HA-tags, in order to directly detect the channels at the surface of fixed oocytes by immunocytochemistry (Figure 5). In these experiments, the plasma membrane was not permeabilized, thus only the extracellular HA-tags of the constructs were measured. The oocytes expressing HA_2_-CD8-TMEM175 (Figure 4E) showed much brighter surface fluorescence after the treatment with dyngo-4a (10 μM, 20 h) than after the application of the vehicle DMSO (Figure 5). This visually confirms the higher amount of TMEM175 channels on the cell surface.

We have also repeated this experiment with the other construct containing the extracellular HA-tag in another position (loopHA-TMEM175, Figure 4H). Identical results were obtained, the abundance of loopHA-TMEM175 in the plasma membrane was also significantly increased by dyngo-4a (supplementary Figure S5). Furthermore, we have also characterized the HA_2_-TMEM175 construct containing cytoplasmic HA-tags (Figure 4A) with and without the permeabilization of the plasma membrane. Intense immunofluorescence only appeared after the permeabilization in the case of HA_2_-TMEM175, since the cytoplasmic HA-tags of the TMEM175 channels in the plasma membrane and intracellular compartments became accessible by the antibodies (supplementary Figure S6).

### 2.5. TMEM175 Current Rapidly Decreases after the Withdrawal of Dynasore

**Figure 6:**
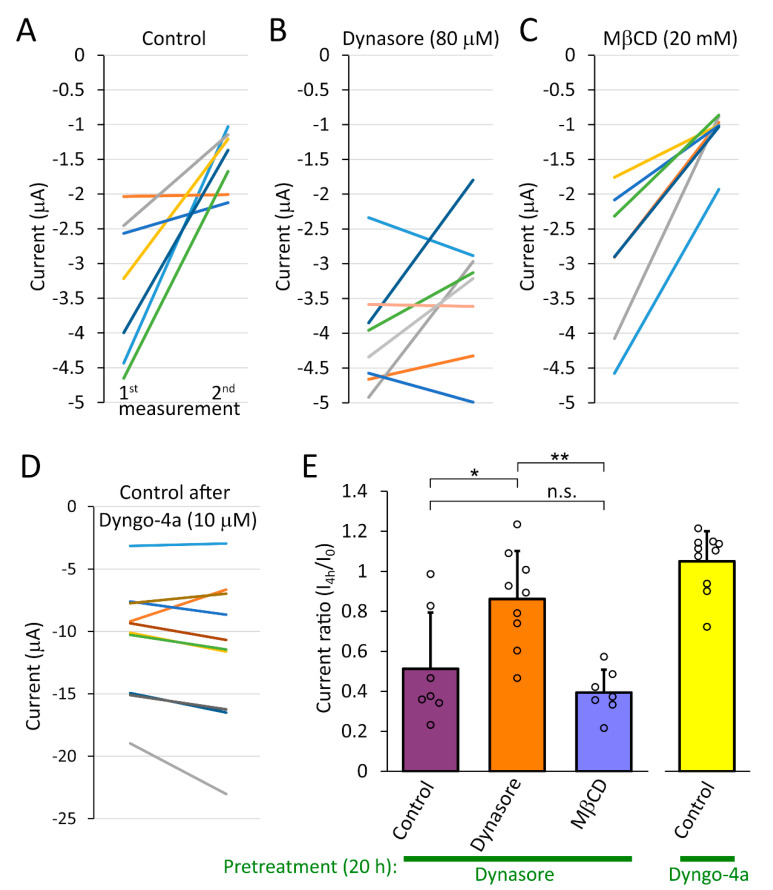
**TMEM175 current rapidly decreases after the withdrawal of dynasore.** (**A**–**C**). Mouse TMEM175 K^+^ current was measured in *Xenopus* oocytes after 20 h treatment with 80 μM dynasore (*1st measurement*). The cells were subsequently incubated under different conditions for 4 h: in control medium ((**A**). *Control*), in the continued presence of 80 μM Dynasore ((**B**). *Dynasore*), or in the presence of 20 mM methyl-β-cyclodextrin ((**C**). *MβCD*), and finally, the K^+^ current was measured again in the same cell (*2nd measurement*). Each colored line interconnects the two current values of a single cell. Two lines nearly overlap in (**C**). (**D**). TMEM175-expressing oocytes were treated overnight with 10 µM dyngo-4a, and subsequently the K^+^ currents were measured twice as in *panel A*, before and 3.5 h after the withdrawal of the inhibitor (i.e., after the incubation in control medium, *Control*). (**E**). Statistical analysis of the data shown in *panels A, B, C, and D*. The currents were normalized to the initial value at the *1st measurement (Current ratio, I_4h_/I_0_)*. One cell with high TMEM175 expression (9.9 to 8.8 μA) in the *Dynasore* group is shown only here, but not in *panel B*. * *p* < 0.02, ** *p* < 0.002 (one-way ANOVA, Tukey HSD test).

TMEM175 current decreased to about half of its initial value in four hours, after the withdrawal of dynasore (80 μM, Figure 6A,E *left column*). In contrast, the current did not decay in the maintained presence of the inhibitor (Figure 6B, compare the *first* and *second* *columns* is Figure 6E). If dynasore increases TMEM175 current predominantly via enhanced surface expression, then the simplest explanation of these data is that an efficient internalization mechanism removes TMEM175 channels from the plasma membrane. If dynasore also increases TMEM175 channel activity in the plasma membrane, then the recovery of the channels to the resting state may also contribute to the effect.

Methyl-β-cyclodextrin (MβCD, 20 mM), the inhibitor of caveolin-dependent endocytosis did not attenuate the reduction of TMEM175 current after the withdrawal of dynasore, suggesting that the caveolin-dependent endocytosis pathway does not contribute to TMEM175 internalization (Figure 6C, *third column* in Figure 6E). In contrast to the rapid decrease of TMEM175 current after the removal of dynasore, TMEM175 current remained stable in the following 4 h after the withdrawal of dyngo-4a (10 μM, applied for 20 h before the withdrawal), possibly because of the accumulation of the high affinity inhibitor in *Xenopus* oocytes (Figure 6D,E *right column*).

### 2.6. Dynasore Compounds More Efficiently Induce TMEM175 Current in the Plasma Membrane Than the Coexpression of Dominant-Negative Dynamin Constructs

The most straightforward explanation for the effect of dynasore on TMEM175 would be that dynasore inhibits dynamin, prevents the clathrin-dependent endocytosis of the channel, and thus the K^+^ current in the plasma membrane is increased. However, solid experimental evidence in favor of this hypothesis has never been provided, as detailed below. The robust effect of the dynasore compounds on TMEM175 current was not mimicked by the coexpression of the dominant-negative dynamin constructs.

The coexpression of K44A mutant dominant-negative rat dynamin-2 (Dyn2-K44A) with TMEM175 resulted in 0.49 ± 0.29 μA Cs^+^ current (*n* = 7), whereas the current was 0.17 ± 0.03 nA in the control group coexpressing wild type dynamin-2 with the channel (*n* = 5, *p* = 0.044, heteroscedastic *t*-test). In another cell preparation, the coexpression of K44A mutant dominant-negative mouse dynamin-3 (Dyn3-K44A) resulted in 1.20 ± 0.87 μA Cs^+^ current (*n* = 9), whereas in the wild type group (Dyn3-WT) the current was 0.41 ± 0.24 μA (*n* = 10, *p* < 0.005, Mann–Whitney U-test). Although statistically significant, the increase was close to the range of endogenous oocyte currents, much smaller than after the treatment with dynasore.

**Figure 7:**
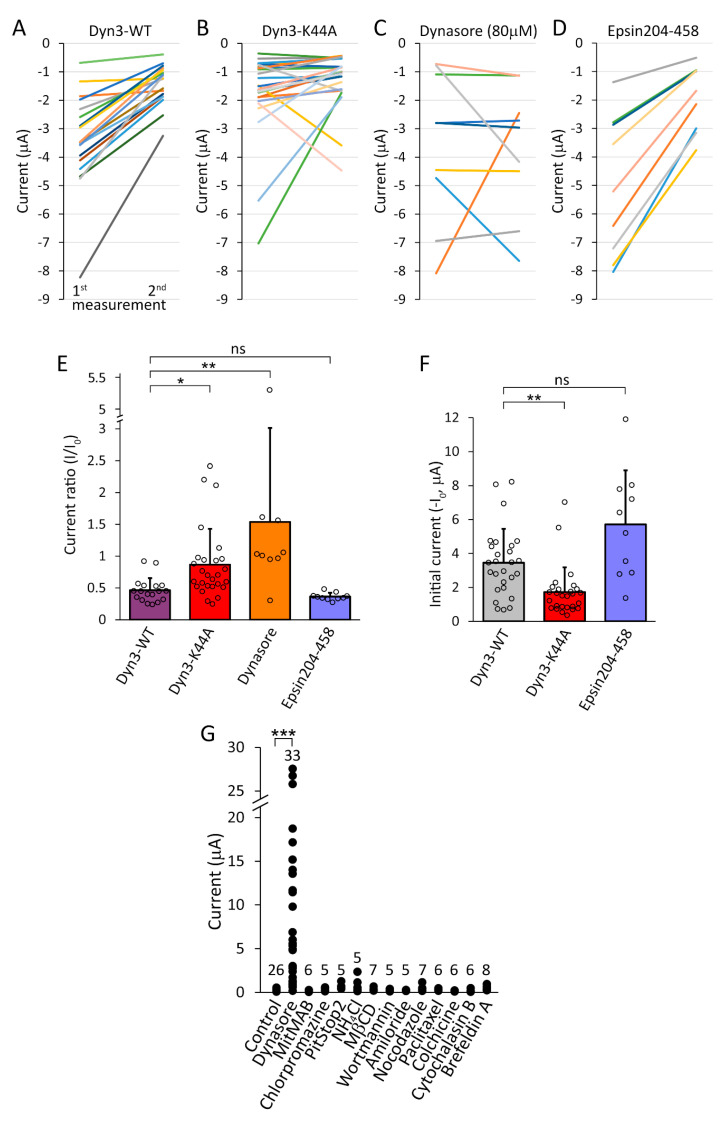
**The effects of dominant-negative dynamin coexpression and different inhibitors of endocytosis on the TMEM175 current.** (**A**–**F**). The effects of dominant-negative dynamin coexpression on the TMEM175 current induced by dynasore, and the decay of this current after the withdrawal of the inhibitor, were examined. The degree of TMEM175 current reduction was measured in four groups of oocytes treated with dynasore (80 μM, 20 h), using the protocol introduced in Figure 6. The cells were incubated for 4 h in the absence or presence of dynasore before the second measurement of TMEM175 current. Each colored line indicates the current change in a single cell. The oocytes derived from two preparations. (**A**). In the negative control group (*Dyn3-WT*), TMEM175 was coexpressed with wild type dynamin-3, and dynasore was absent between the two measurements of the K^+^ current. (**B**). In this group (*Dyn3-K44A*), TMEM175 was coexpressed with dominant-negative K44A mutant dynamin-3, and dynasore was absent between the two measurements. (**C**). In the positive control group (*Dynasore (80 μM)*), TMEM175 was coexpressed with wild type dynamin-3, however, dynasore was present between the two measurements. (**D**). In this group (*Epsin204-458*), TMEM175 was coexpressed with the clathrin-mediated endocytosis inhibitor DPW domain of epsin, and dynasore was absent between the two measurements. (**E**,**F**). Statistical analysis of the data shown in *panels A–D*. The currents were normalized to the initial value at the *1st measurement (Current ratio, I/I_0_*, (**E**)) or the initial current amplitude (−I_0_ at the *1st measurement*) is plotted (**F**). Current data of the *1st measurements* from *panels A and C* are pooled for the *Dyn3-WT* group of *panel F (first grey column)*. One cell with high TMEM175 expression (11.9 to 4.3 μA) in the *Epsin204-458* group is shown only in *panel E and F,* but not in *D*. The omission of the data point of 5.34-fold current increase from the *Dynasore (80 μM)* group would not affect the statistically significant differences in this experiment. * *p* < 0.02, ** *p* < 0.002 (multiple comparisons after Kruskal–Wallis ANOVA), ns: not significant. (**G**). Different inhibitors of endocytosis and modulators of cytoskeleton do not induce TMEM175 current in the plasma membrane. The oocytes expressing mouse TMEM175 were treated with the different pharmacological agents, as indicated *below the graph*. Each point represents a measured cell, and the number of cells is shown above the data points. The oocytes derived from six preparations and the control and dynasore-treated cells were measured from each preparation. Control cells were incubated without the active compounds, in the presence of the vehicle (DMSO), where appropriate. The applied concentrations were the following: Dynasore (80 μM), MitMAB (tetradecyl-trimethyl-ammonium-bromide, 15 μM), Chlorpromazine (50 μM), PitStop2 (40 μM), NH_4_Cl (15 mM ^#^), MβCD (Methyl-β-cyclodextrin, 40 mM ^#^), Wortmannin (1 μM), Amiloride (1 mM), Nocodazole (1 μM), Paclitaxel (50 nM), Colchicine (10 μM), Cytochalasin B (10 μM), Brefeldin A (5 μM). (^#^ the osmotic concentration was higher during these treatments.) The compounds were applied overnight (for 20 h). *** *p* < 10^−6^ (Kruskal–Wallis ANOVA).

By taking advantage of the method of repeated recordings introduced in Figure 6, the effect of Dyn3-K44A on the kinetics of TMEM175 internalization after the withdrawal of dynasore has also been examined (Figure 7). In the group coexpressing dominant-negative *Dyn3-K44A* with TMEM175, the K^+^ current decreased relatively less in four hours (87 ± 56 % remaining current, *n* = 27, Figure 7B,E *red column*) than in the control *Dyn3-WT* group (46 ± 19 %, *n* = 18, Figure 7A,E *purple column*, *p* < 0.02). The large relative remaining current in the *Dyn3-K44A* group would suggest that TMEM175 internalization is inhibited by dominant-negative dynamin. However, Dyn3-K44A also reduced the initial K^+^ current evoked by the overnight application of dynasore, compared to Dyn3-WT (*1st measurement* in Figure 7A (plus C) versus B, *left pair of columns* in Figure 7F, *p* < 0.002). Therefore, Dyn3-K44A interferes with the expression or plasma membrane insertion of TMEM175, and the reduction of the initial current in the *Dyn3-K44A* group prevents the realistic comparison of the recovery kinetics of TMEM175 current between the *Dyn3-WT* and *Dyn3-K44A* groups. Altogether, these results do not confirm the substantial contribution of dynamin to the increased TMEM175 current. It is evident that Dyn3-K44A exerts qualitatively different effects on TMEM175 from dynasore, and Dyn3-K44A is much less effective than the dynasore compounds.

The DPW motifs of epsin avidly bind to the adapter protein complex 2 (AP-2), and induce dominant-negative effect on clathrin-mediated endocytosis [26]. The current decay after the withdrawal of dynasore was not affected by the coexpression of Epsin204-458 with TMEM175 (Figure 7D,E), arguing against the role of clathrin-mediated endocytosis in TMEM175 internalization.

### 2.7. Several Inhibitors of Endocytosis Fail to Mimic the Action of Dynasore

Dynasore is a potent inhibitor of dynamin and the process of endocytosis [15]. However, other widely used inhibitors of endocytosis failed to induce TMEM175 current in the plasma membrane (Figure 7G). MitMAB (tetradecyl-trimethyl-ammonium-bromide), another dynamin inhibitor [27], and the nonselective inhibitors of clathrin-dependent endocytosis, chlorpromazine and PitStop2 [28,29] were not effective. TMEM175 current was not increased by the lysosomotropic NH_4_Cl [30], or the generally used inhibitor of caveolin-dependent endocytosis, methyl-β-cyclodextrin (MβCD), which was reported to deplete cholesterol from the plasma membrane of *Xenopus* oocytes [31,32,33]. High concentrations of wortmannin or amiloride, inhibitors of pinocytosis [34], also did not enhance the functional expression of TMEM175. The microtubule-depolymerizing nocodazole and colchicine, the microtubule-stabilizing paclitaxel, the actin polymerization inhibitor cytochalasin B, and the disruption of the Golgi apparatus with brefeldin A did not induce K^+^ current in the plasma membrane [35]. Another type of actin polymerization inhibitor, latrunculin A also did not increase TMEM175 current in the plasma membrane (supplementary Figure S7). In summary, none of the additionally tested chemicals were effective (Figure 7G.), suggesting that the pharmacological profile of dynasore is specifically suited for the functional expression of TMEM175 in the plasma membrane.

### 2.8. TMEM175 Current through the Plasma Membrane Is Regulated by Protein Kinase B (PKB)

**Figure 8:**
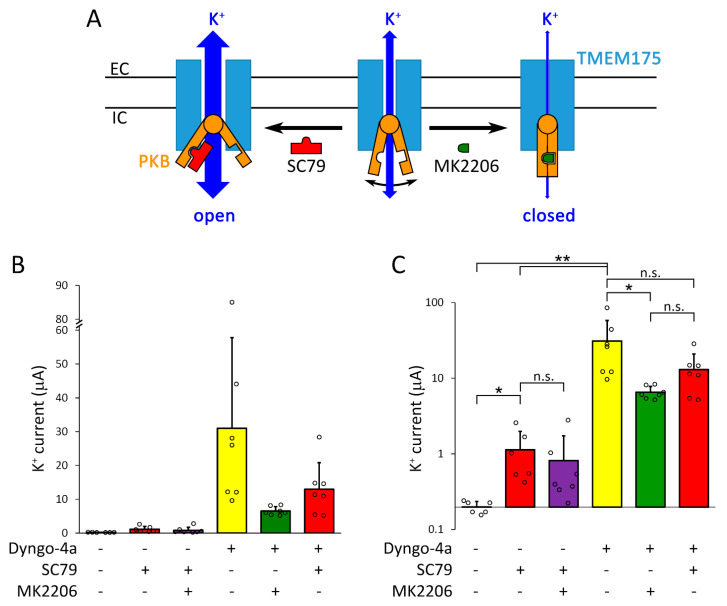
**The effects of SC79, MK2206 and dyngo-4a on TMEM175 current.** (**A**). Schematic illustration of TMEM175 regulation by the binding of SC79 or MK2206 to protein kinase B (PKB). (**B**). The oocytes expressing TMEM175 were incubated in the presence or absence of dyngo-4a (10 μM), SC79 (20 μM), and MK2206 (20 μM) for 20 h, as indicated *below the graph*. TMEM175 inward currents were measured in 80 mM K^+^ at −100 mV. (**C**). The same data as in *panel B* are plotted on a logarithmic scale to clearly visualize the differences among the groups. Statistical analysis was performed on log-transformed data. * *p* < 0.05, ** *p* < 0.001 (Welch’s ANOVA, Dunnett T3 post hoc test), n.s.: not significant.

It has recently been described that PKB directly binds to TMEM175, the channel is opened by the conformational changes of PKB, and the regulation does not require the enzymatic activity of the kinase [14]. The PKB activator SC79, and allosteric PKB inhibitor MK2206 were reported to change TMEM175 current via the interaction with PKB associated to the channel [14]. SC79 binds to the pleckstrin homology (PH) domain of PKB, stabilizes the active ‘open’ conformation of the kinase, and causes channel opening via the conformational coupling between the two proteins (for schematic illustration, Figure 8A) [14]. PKB was also reported to enhance the plasma membrane insertion of different ion channel types, including potassium channels [36,37,38].

Therefore, we investigated whether PKB regulates TMEM175 in the plasma membrane, in the absence or presence of dyngo-4a. The PKB activator SC79 (20 μM, 20 h) increased the K^+^ current in the plasma membrane of TMEM175-expressing oocytes to about 1 μA in the absence of dyngo-4a (*p* < 0.05, compared to the untreated control cells expressing TMEM175, Figure 8B,C, *first* vs. *second columns*). MK2206 did not significantly inhibit the small (≈1 μA) current induced by SC79 (Figure 8B,C, *second* and *third* columns), suggesting that the activation of PKB (and TMEM175 current) by SC79 prevailed over the inhibition by MK2206. Although we did not examine the mechanism of action of SC79, the data are consistent with the conclusion that SC79 slightly increases TMEM175 current in the plasma membrane, in good accordance with the previously proposed model.

In contrast to SC79, dyngo-4a (10 μM, 20 h) resulted in large (about 30 μA) current on average in the same cell preparation (Figure 8B,C, *second* vs. *fourth columns*, *p* < 0.001, for the precise average ± S.D. values, see supplementary Figure S8). MK2206 (20 μM, 20 h) allosteric PKB inhibitor caused about 80% inhibition of TMEM175 current induced by dyngo-4a (Figure 8B,C, *fourth* vs. *fifth columns*, *p* < 0.05). Based on the reported model [14], MK2206 inhibits TMEM175, since it simultaneously binds to the PH and near the catalytic domain of PKB and stabilizes the inactive ‘closed’ conformation of the kinase and (consequently) the channel (Figure 8A). The substantial inhibition of TMEM175 current by MK2206 in the presence of dyngo-4a may be caused by the inhibition of the active TMEM175-PKB complex by MK2206 in the plasma membrane, or by the interference of MK2206 with the effect of dyngo-4a. Irrespective of the mechanism, the efficiency of MK2206 confirms independently from SC79 that TMEM175 current in the plasma membrane is regulated by PKB.

SC79 did not increase the current induced by dyngo-4a; in fact, there was a tendency for inhibition (however, this effect was not statistically significant, Figure 8B,C, *fourth* and *sixth columns*). SC79 increased TMEM175 current in the absence, but not in the presence of dyngo-4a, suggesting that TMEM175-PKB complex in the plasma membrane was in the active state after the application of the dynasore compound.

**Figure 9:**
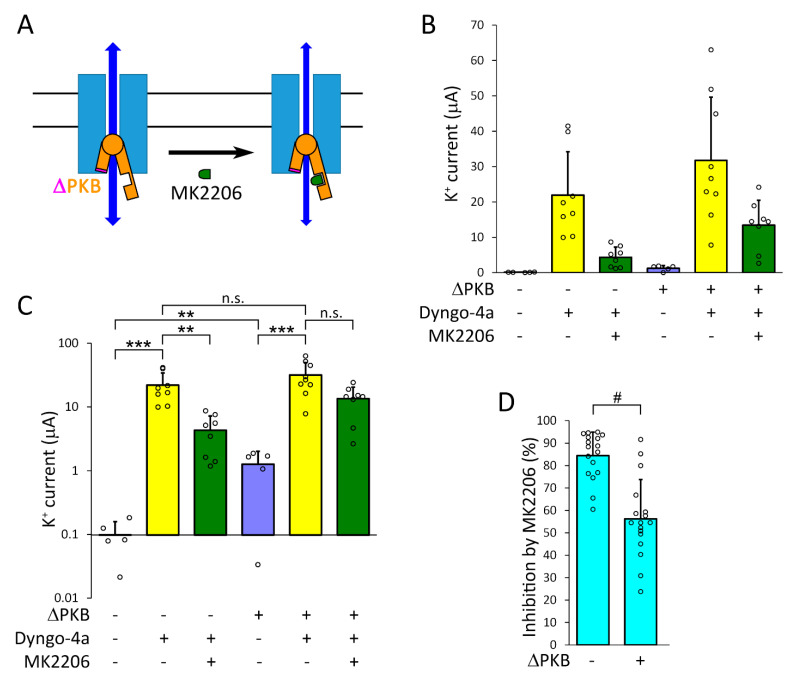
**The coexpression of the constitutively active, PH domain deleted protein kinase B (ΔPKB) construct with TMEM175 results in small K^+^ currents, and reduces the MK2206-mediated inhibition of the TMEM175 current induced by dyngo-4a.** (**A**). Schematic illustration that the degree of TMEM175 current inhibition by MK2206 is reduced, when full length protein kinase B is replaced by ΔPKB in the TMEM175-PKB protein complex. (**B**). The oocytes expressing TMEM175 alone, or coexpressing the channel with ΔPKB, were treated with dyngo-4a (10 μM) or dyngo-4a plus MK2206 (20 μM) for 20 h, as indicated *below the graph*. TMEM175 inward currents were measured in 80 mM K^+^ at −100 mV. (**C**). The same data as in *panel B* are plotted on a logarithmic scale to visualize the differences among the groups. Statistical analysis was performed on log-transformed data. ** *p* < 0.005, *** *p* < 0.0005 (ANOVA, Tukey HSD post hoc test), n.s.: not significant. (**D**). Relative inhibition of TMEM175 K^+^ current by MK2206 was determined in the absence or presence of ΔPKB, as indicated *below the graph*. The experiment illustrated in *panel B* was repeated in another oocyte preparation with identical results, and the data points of the currents inhibited by MK2206 (*data points of the green columns*) were normalized to the average current in the absence of MK2206 (*yellow columns*). Note that the coexpression of ΔPKB reduced the inhibition of TMEM175 current by MK2206. # *p* < 10^−4^ (Mann–Whitney U-test).

In order to confirm the above conclusions by using molecular biological methods in addition to pharmacology, we have cloned a constitutively active version of mouse PKB with deleted PH domain (ΔPKB). According to the reported mechanism of action of MK2206 (Figure 8A) [14], the inhibition of PKB by MK2206 can be reduced by the deletion of the PH domain of the kinase, when one of the binding sites of MK2206 is eliminated, and the inhibitor does not stabilize the closed conformation of the enzyme (Figure 9A). The coexpression of ΔPKB increased TMEM175 current in the plasma membrane to about 1 μA in the absence of dyngo-4a (*p* < 0.005, compared to the control cells expressing only TMEM175, Figure 9B,C, *first* and *fourth columns*). Thus, ΔPKB (similarly to SC79) significantly increased TMEM175 current in the plasma membrane, confirming the role of the kinase in the regulation of the channel.

Dyngo-4a (10 μM, 20 h) robustly increased TMEM175 current both in the absence (Figure 9B,C, *first* and *second columns*, *p* < 0.0005) and presence of ΔPKB (Figure 9B,C, *fourth* and *fifth columns*, *p* < 0.0005). The coexpression of ΔPKB with TMEM175 did not significantly increase the K^+^ current measured after the treatment of the cells with dyngo-4a (10 μM, 20 h, Figure 9B,C, *second* vs. *fifth columns*), similarly to the inefficiency of SC79 in the presence of dyngo-4a.

MK2206 inhibited TMEM175 current by about 85% in the absence of ΔPKB, when only the endogenous PKB of the oocyte formed complex with the channel (Figure 9B,C, *second* and *third columns*, *p* < 0.005). However, the degree of inhibition of TMEM175 current by MK2206 was reduced to about 55% in the presence of ΔPKB (Figure 9B,C, *fifth* vs. *sixth columns*, the inhibition of TMEM175 by MK2206 decreased to a level, which did not reach statistical significance). The degrees of inhibition by MK2206 in the absence and presence of ΔPKB were significantly different (for statistical analysis, see Figure 9D, *p* < 10^−4^). This indicates that ΔPKB was functionally expressed, regulated TMEM175, and a major part of the effect of MK2206 was mediated by the inhibition of PKB.

## 3. Discussion

The association of single nucleotide polymorphisms (SNPs) in the GAK/DGKQ locus on chromosome 4 with Parkinson’s disease raised the possibility that TMEM175 gene in this region has pathophysiological significance in the neurodegenerative disorder [1]. The role of TMEM175 sequence variants in the development and age of onset of Parkinson’s disease was further confirmed by several recent studies [2,3,4,5,6,7]. TMEM175 coding variant p.M393T is among the four strongest common genetic risk factors for Parkinson’s disease [4,5,8,9]. The other three high-risk genes encode α-synuclein (SNCA), glucocerebrosidase (GBA), and leucine-rich repeat kinase 2 (LRRK2). These genes are related to the lysosomal decomposition of phosphorylated α-synuclein, which is a major constituent of Lewy bodies, the pathological hallmark of Parkinson’s disease [39,40,41,42,43,44,45,46].

Potassium permeability of the lysosomal membrane and the dependence of the vesicular membrane potential on the cytoplasmic K^+^ concentration were diminished in a TMEM175 knock-out cell line [10]. TMEM175 has also been shown to contribute to lysosomal pH stabilization, most probably by providing appropriate charge compensation when the proton transport through the vesicular membrane is stimulated. Accordingly, if TMEM175 is deleted or carries the pathogenic M393T mutation, the lysosomal lumen becomes too alkaline in the case of nutrient starvation of the cells, when autophagy is intensified [10,47]. The unstable lysosomal pH may explain the reduced glucocerebrosidase catalytic activity, and leads to impaired lysosomal clearance and accumulation of phosphorylated α-synuclein [21,47].

The molecular architecture of TMEM175 is substantially different from the other typical potassium channels of the plasma membrane. In most potassium channels, the high K^+^ selectivity is determined by the structure of the selectivity filter, which is composed of the conserved pore (P) loops with the TVGYG-like signature sequences. Although human TMEM175 is highly selective for potassium, about 20–40-fold over Na^+^, it does not contain the P loop [10]. Atomic resolution structures of human TMEM175 provided insight into the alternative mechanisms of K^+^ selectivity [11,12,13]. Mammalian TMEM175 subunits contain two repeats of six-transmembrane-segment domains in a single polypeptide chain (altogether 12 TMS/subunit, Figure 4A), and the channel functions as the homodimer of subunits (24 TMS/channel). The protein complex possesses a transmembrane ion-conduction pore in its central axis, formed by the amino acid side chains of TM1 and TM7 pore-lining helices. Highly conserved rings of isoleucine [11,12], or threonine and serine residues [13] around the axis of the pore, have been reported to be the structural determinants of K^+^ selectivity.

In this study, we report for the first time that mouse TMEM175 lysosomal K^+^ channels are functionally expressed in the plasma membrane of *Xenopus laevis* oocytes in response to the treatment of the cells with dynasore or dyngo-4a. This conclusion is unequivocally supported by the experimental evidence that the 4-AP-sensitive Cs^+^ current through the plasma membrane is increased to very high levels in the oocytes injected with TMEM175 cRNA, *and* treated with the dynasore compounds. In the absence of the treatment with dynasore, the identical injection of TMEM175 cRNA resulted in negligible Cs^+^ currents in the range of the endogenous conductance of the oocytes. Similarly, the Cs^+^ currents were absent when non-injected oocytes were treated with dynasore, indicating that TMEM175 expression is required for the effect.

The dynasore compounds are highly efficient inhibitors of dynamin GTPase activity, and interfere with the clathrin-dependent endocytosis by blocking the transition from fully formed pits to pinched-off vesicles [15,16]. The hydroxylated dynasore derivative, dyngo-4a more potently inhibits dynamin than the parent compound. In accordance with its high potency for dynamin inhibition, dyngo-4a induced more prominent TMEM175 currents in the plasma membrane than dynasore, and the EC_50_ values were consistent with the contribution of dynamin to the effect. In an effort to evaluate the role of dynamin, we coexpressed different dominant-negative dynamin constructs with TMEM175. Unexpectedly, the overexpression of Dyn2-K44A and Dyn3-K44A only slightly increased TMEM175 current, much less than the application of the dynasore compounds. Although Dyn3-K44A slowed down the decay of TMEM175 current after the withdrawal of dynasore, it also reduced the initial current induced by dynasore more efficiently than the wild type dynamin-3. These data suggest that the elimination of dynamin function is not equivalent to the action of dynasore compounds, and raise the possibility that dynamin-independent effects also contribute to the increase of TMEM175 current in the plasma membrane. The higher potency of dyngo-4a than dynasore points to the contribution of another dynamin-like GTPase, however, the presently known alternative GTPase targets of dynasore compounds, e.g., the mitochondrial Drp1 [15], or atlastins [48], are not probable mediators of the effect on TMEM175.

The dynasore compounds can increase TMEM175 current via two not mutually exclusive mechanisms, by the accumulation of channels in the plasma membrane or by the activation of the channels already present at this location. Immunocytochemistry data indicate that the number of TMEM175 channels is increased by dyngo-4a in the plasma membrane. The semiquantitative nature of Western blot and on-cell surface luminometry experiments prevented the determination, how many times the number of TMEM175 channels is increased by dyngo-4a. Whereas the precise measurement of small TMEM175 currents is possible by the two-electrode voltage clamp method, the exact quantitation of the small decrease of anti-HA signal by proteinase K on the immunoblot is problematic in the case of control DMSO-treated oocytes. Similarly, the on-cell surface anti-HA signals of the extracellularly tagged TMEM175 constructs in the DMSO group were in the range of the background luminescence of ECL reagent itself, or the detector noise of the confocal microscope. Because of these technical limitations, it remains an open question whether the enhanced surface expression of TMEM175 is fully responsible for the increase of the K^+^ current, or additional channel activation also contributes to the effect.

The steady-state level of TMEM175 protein on the cell surface can be increased by the acceleration of channel insertion into, or the decreased rate of internalization from the plasma membrane (or both). As the first approximation to this question, we measured the kinetics of decay of TMEM175 current after the withdrawal of dynasore. TMEM175 current decreased with a rapid kinetics (t_1/2_ ≈ 4 h). This time constant (half-life of decay) itself is not necessarily characteristic for the biological phenomenon, since it may simply represent the kinetics of dynasore washout from the oocyte. However, the experimentally determined t_1/2_ provides an upper estimate for the real half-life of TMEM175 current, which could be measured after the prompt removal of the inhibitor. If dynasore acts predominantly by increasing the number of channels on the cell surface, then this half-life of decay indicates that TMEM175 is internalized from the plasma membrane by a rapid and highly efficient mechanism, irrespective of whether the pharmacological effect of dynasore is the acceleration of channel insertion or the inhibition of the internalization process.

The inhibition of dynamin generally interferes with the clathrin-dependent endocytosis, and internalization processes [49]. However, we found that the pharmacological inhibition of the clathrin-dependent pathway by chlorpromazine, PitStop2, and the coexpression of the dominant-negative epsin construct did not induce TMEM175 current in the plasma membrane, and did not affect the kinetics of decrease of TMEM175 current after the withdrawal of dynasore. Dynamin also has a pivotal role in clathrin-independent mechanisms of endocytosis [50], however, the inhibition of the caveolin-dependent pathway with methyl-β-cyclodextrin, and the application of wortmannin or amiloride to block pinocytosis, did not result in the appearance of TMEM175 current. It is a possible explanation that the inhibition of endocytosis by the above procedures was not complete, and the remaining activity was sufficient for TMEM175 internalization. Alternatively, TMEM175 may be internalized by redundant pathways or via a less well-studied endocytosis mechanism.

It is well established that dynasore evokes off-target (dynamin-independent) effects related to endocytosis. Dynasore reduces the cholesterol level in the plasma membrane, and disrupts lipid rafts independently of its action on dynamin [51]. Furthermore, dynasore and dyngo-4a efficiently inhibit fluid-phase endocytosis and membrane ruffling in dynamin triple-knock-out fibroblasts, indicating that dynasore compounds also interfere with less well-characterized dynamin-independent endocytosis processes [52]. Irrespective of the mechanism of action of dynasore for the induction of TMEM175 current in the plasma membrane, it is evident from our data that the generally used inhibitors of endocytosis do not evoke a similar effect as the dynasore compounds under identical conditions.

It has recently been reported that TMEM175 is regulated by protein kinase B [14]. The N-terminal pleckstrin homology (PH) domain of active PKB binds phosphatidylinositol-3,4,5-triphosphate (PIP_3_) in the plasma membrane under physiological conditions and perhaps phosphatidylinositol-3,4-biphosphate or phosphatidylinositol-3,5-biphosphate (PtdIns(3,4)P_2_ or PtdIns(3,5)P_2_) in the lysosomal membranes [53,54,55]. The opening of the kinase structure by the release of the PH domain from the catalytic domain activates TMEM175 [14]. SC79 pharmacologically stabilizes PKB open conformation by binding to the PH domain, whereas the allosteric PKB inhibitor MK2206 closes the structure by simultaneously associating to the PH and near the catalytic domain (Figure 8A).

Dyngo-4a induced much (around 20-fold) larger TMEM175 currents than the PKB activator SC79, or the coexpression of the constitutively active ΔPKB with the channel. The treatment with SC79 efficiently activated PKB, since the (small, ≈1 μA) TMEM175 current in the presence of SC79 was not significantly affected by the PKB inhibitor MK2206. In turn, MK2206 efficiently inhibited PKB (in the absence of SC79), because it decreased TMEM175 current by about 80–85% in the presence of dyngo-4a. If ΔPKB was coexpressed with the channel, MK2206 exerted less inhibition on the TMEM175 current induced by dyngo-4a (only about 55% inhibition), suggesting that ΔPKB was functionally expressed and the deletion of its PH domain interfered with the known mechanism of action of MK2206 [56,57].

The TMEM175 channels, which were translocated into the plasma membrane by dyngo-4a, were not further activated by SC79. In fact, SC79 showed the tendency for inhibition of the dyngo-4a-induced TMEM175 current (about 50% inhibition, the effect was not significant), which may be caused by the direct inhibition of the channel by the high concentration of the PKB activator, or the pharmacological interference of SC79 with the action of dyngo-4a. This inhibitory tendency was not apparent in the experiments with ΔPKB. The coexpression of ΔPKB with TMEM175 showed the tendency for activation of the dyngo-4a-induced current (about 50% activation, not significant). Considering these data together, we summarize that the activation or overexpression of PKB did not significantly change the dyngo-4a-induced TMEM175 current. The unaltered TMEM175 current by PKB activation, together with the statistically significant robust inhibition of the same current by the PKB inhibitor MK2206 suggest that TMEM175 was already in the active state after the treatment with dyngo-4a, even in the absence of growth factors in the incubation medium of the oocytes.

We report for the first time that TMEM175 channels can be redirected to the plasma membrane with a simple pharmacological treatment. As this occurs in a heterologous expression system, it raises concerns whether robust overexpression of TMEM175 is required for the plasma membrane insertion. It is an advantage of the *Xenopus* oocyte system that the expression levels can be adjusted by the amount of the injected cRNA. We determined the dose-response relationship between cRNA quantity and dyngo-4a-induced K^+^ current, and established that 0.5–1.5 ng/oocyte TMEM175 cRNA may be enough to obtain currents in the low μA range. In our experience, similar cRNA quantities are required for the expression of several other K^+^ channel types, which are normally targeted to the plasma membrane (although there is substantial variation in this respect among the different types). Thus, we think that extreme overexpression of TMEM175 is not required for the current in the plasma membrane.

Occasionally, the underlying (patho)physiology is discovered later than the corresponding pharmacological effects. In this case, it is tempting to speculate that the cell possesses the appropriate molecular machinery for the insertion of TMEM175 into, and the retrieval of this channel from the plasma membrane, and a simple pharmacological trigger is enough to change the localization. This raises the idea that TMEM175 may also appear in the plasma membrane of native cells under certain conditions. Newly synthesized TMEM175 protein may be first translocated to the plasma membrane, followed by its recovery into the endosomes, as the normal pathway under physiological conditions. Alternatively, the internalization may be a protective mechanism against the pathological mislocalization of the channel. In the plasma membrane, TMEM175 is a fully functional background K^+^ channel; in sufficient quantity, it induces hyperpolarization and reduces the cellular excitability. It remains to be examined whether TMEM175 is present in the plasma membrane of native cells, e.g., in the dopaminergic neurons of the substantia nigra pars compacta under the pathological conditions of Parkinson’s disease. It may require substantially different methodology from those applied in the present study to investigate this challenging problem [58,59,60,61,62], and identify the 4-AP-sensitive Cs^+^ current of TMEM175 channels at −100 mV in the plasma membrane of native cells.

## 4. Conclusions

The expression of mouse TMEM175 results in very small currents through the plasma membrane in *Xenopus laevis* oocytes, in good accordance with the previously reported endosomal/lysosomal localization of this K^+^ channel type. Unexpectedly, however, after the treatment of the cells with the dynamin inhibitor dynasore compounds, robust TMEM175 K^+^ currents can be measured in the plasma membrane with the two-electrode voltage clamp method. The hydroxylated derivative dyngo-4a more efficiently induces TMEM175 surface expression than the parent compound dynasore. TMEM175 currents in the plasma membrane can be more easily investigated by conventional electrophysiological techniques than the patch clamping of intracellular organelles. TMEM175 ion selectivity, sensitivity to 4-aminopyridine (4-AP), and regulation by protein kinase B (PKB) are similar in the plasma membrane as those previously reported in the lysosomes. Thus, the overall functional characteristics of TMEM175 are maintained in the pharmacologically induced plasma membrane localization.

The coexpression of dominant-negative dynamin with the channel, or the application of different inhibitors of endocytosis were by far less effective on TMEM175 than the application of the dynasore compounds. This suggests that dynamin-independent pharmacological effects of the dynasore compounds may also contribute to the mechanism of action, and the pharmacological profile of dyngo-4a is especially suited for the induction of TMEM175 current in the plasma membrane.

## 5. Materials and Methods

### 5.1. Materials

Chemicals of analytical grade were purchased from Sigma (St. Louis, MO, USA), Fluka (Milwaukee, WI, USA), Enzo Life Sciences (Farmingdale, NY, USA), Santa Cruz Biotechnology (Dallas, TX, USA) or Merck (Whitehouse Station, NJ, USA). Enzymes and kits for molecular biology applications were purchased from Ambion (Austin, TX, USA), QIAGEN (Hilden, Germany), Thermo Fisher Scientific (Waltham, MA, USA), New England Biolabs (Beverly, MA, USA) and Stratagene (La Jolla, CA, USA). Dynasore and dyngo-4a (Dynamin Inhibitor IV, Hydroxy-Dynasore, Calbiochem, Sigma-Aldrich/Merck #324413) were prepared as stock solutions in DMSO (5 mg/mL and 10 mg/mL, respectively). All other chemicals were dissolved in DMSO or water, as recommended by the manufacturer, and the final DMSO concentration was always less than 2%, in most experiments < 0.5%. The stock solutions were stored at −20 °C, and diluted further before the application.

### 5.2. Molecular Biology

TMEM175 cDNA was amplified by PCR using Pfu polymerase (Thermo Fisher Scientific) after the reverse transcription (RevertAid Reverse Transcriptase, Thermo Fisher Scientific) of total RNA purified from mouse testis with TRIzol reagent (Invitrogen, Carlsbad, CA). The primers were 5′ CAGGAATTCGCCGCCACCATGTCCAGGCTCCAGACTG 3′ and 5′ GCGCTCGAGTTAGCAGGGGTCAGGCAAGAGC 3′, forward and reverse, respectively. TMEM175 PCR product was cloned into pXEN vector (GenBank: EU267939.1) at the EcoRI-XhoI sites. The TMEM175 coding sequence was amplified from the above construct with Phusion polymerase (Thermo Fisher Scientific), using the above forward and the 5′ GGTAACCTGATCATTAGCAGGGGTCAGGCAAGAGC 3′ reverse primer, the PCR product was cleaved with the EcoRI and BclI enzymes, and subcloned into the pIRES-CD8 vector (Michel Lazdunski’s laboratory, CNRS Institute of Molecular and Cellular Pharmacology, Sophia Antipolis, France, [63]) at the EcoRI-BamHI sites.

In the HA_2_-TMEM175 construct containing the intracellular N-terminal double influenza haemagglutinin (HA) tag, the MGSGYPYDVPDYAGGYPYDVPDYAGQL***MSRLQTEE…*** amino acid sequence was added using PCR-based and oligonucleotide dimer insertion techniques (the HA epitope sequences are *underlined*, the original N-terminus of TMEM175 is indicated in *bold italics*). In the HA_2_-CD8-TMEM175 construct containing the extracellular N-terminal double influenza haemagglutinin (HA) tag and human CD8 transmembrane segment, the MGSGYPYDVPDYAGGYPYDVPDYAGQL*GAVHTR…PVVKSG*FE***RLQTEE…*** amino acid sequence was added (the HA epitope sequences are *underlined*, the CD8 sequence is in *italics*, the original N-terminus of TMEM175 is indicated in *bold italics*). In the loopHA-TMEM175 construct containing the extracellular HA-tag in the fifth EC loop of the channel, the QTSAFARQPHD sequence of TMEM175 was replaced with the HA epitope and flanking glycines GYPYDVPDYAG.

The cDNAs of the HA epitope-tagged wild-type and K44A mutant rat dynamin-2 (*Dyn2-WT* and *Dyn2-K44A*) in pcDNA3 vector were kindly provided by Dr. K. Nakayama (Tsukuba Science City, Ibaraki, Japan, [64,65]). These coding sequences were amplified by the Phusion polymerase with the 5′ CCGGCCAAGCTTGCCGCCACCATGGGCAACCGCGG 3′ and 5′ CGCGCATGCCTAGTCGAGCAGGGACGGC 3′ primers, and subcloned into pXEN vector at the HindIII-PaeI sites. Mouse dynamin 3 (isoform2, GenBank: NM_172646.3) was amplified by RT-PCR from mouse brain RNA with Phusion polymerase (Thermo Fisher Scientific), using the primers 5′ CAGGAATTCGCCGCCACCATGGGGAACCGGGAGATGGAAG 3′ and 5′ GCGAGATCTTTAGTCTAACAGTGAGGATTCTAGTGG 3′, and cloned into pXEN vector at the EcoRI-BglII sites (*Dyn3-WT*). The K44A mutant version of the construct (*Dyn3-K44A*) was produced by the QuikChange in vitro site-directed mutagenesis method (developed by Stratagene), using Phusion polymerase and a complementary primer pair with the sense sequence: 5′ GAGCGCCGGCGCTAGCTCAGTGCTC 3′. Mutant clones were selected by taking advantage of the incorporated NheI restriction enzyme cleavage site. The fragment 204–458 of mouse epsin 1 (GenBank: NM_001252454.1) was amplified by RT-PCR from mouse brain RNA with 5′ CAGGAATTCGCCGCCACCATGTCCTGCGGCCCTGAGGATGAC 3′ forward and 5′ GCGCTCGAGTCATGCAGCAGGTGGGGGACTCC 3′ reverse primers, and cloned into pXEN vector at the EcoRI-XhoI sites (*Epsin204-458*). The cDNA of PKB construct with deleted pleckstrin homology (PH) domain (Δ*PKB*) was amplified by RT-PCR from mouse brain RNA with 5′ CAGGAATTCGCCACCATGGGTTCGAAGGGGGCTGAAGAGATGGAGGTGTC 3′ forward and 5′ ATACCGTCGACTCAGGCTGTGCCACTGGCTGAG 3′ reverse primers, cleaved with EcoRI and SalI enzymes and cloned into pXEN vector at the EcoRI-XhoI sites. In ΔPKB, the N-terminal 129 amino acids of full length PKB were deleted. The sequence of the clones was verified by the sequencing service of Macrogen Europe (Amsterdam, The Netherlands).

The cRNAs coding for TMEM175 and the different dynamin, epsin and PKB constructs were synthesized using the mMESSAGE mMACHINE™ T7 in vitro transcription kit (Ambion, Austin, TX, USA), according to the instructions of the manufacturer. The plasmid templates for the reaction were linearized with XbaI, NotI or SmaI restriction enzymes. The quality of all cRNA preparations were verified by denaturing formaldehyde agarose gel electrophoresis and ethidium bromide staining.

### 5.3. Maintenance of Xenopus laevis and Oocyte Microinjection

*Xenopus* oocytes were prepared as previously described [66]. In order to improve survival, the oocytes with high K^+^ conductance of the plasma membrane were incubated in modified Barth’s solution containing high K^+^ (MB-HK). MB-HK contained (in mM): 66.9 NaCl, 25.8 mM KCl, 1.8 mM NaHCO_3_, 0.62 mM MgSO_4_, 0.25 mM Ca(NO_3_)_2_, 0.31 mM CaCl_2_, 15.2 mM HEPES buffered to pH 7.5 with NaOH and supplemented with penicillin (76 units/mL), streptomycin (76 µg/mL), sodium pyruvate (3.4 mM), and theophylline (0.38 mM). Typically, 2–12 ng cRNA of the TMEM175, dynamin, epsin or ΔPKB constructs was microinjected per oocyte in 50 nL volume with Nanoliter Injector (World Precision Instruments, Saratosa, FL, USA).

Fifteen frogs were used for the experiments. The animals were maintained in two 50 L tanks, with continuous filtering and water circulation through aquarium pumps at 19 °C in an air conditioned room. The frogs were anaesthetized with 0.1% tricaine solution and sacrificed by decerebration and pithing. All treatments of the animals were conducted in accordance with state laws, institutional regulations, and National Institutes of Health guidelines. The experiments were approved by the Animal Care and Ethics Committee of Semmelweis University (approval ID: XIV-I-001/2154-4).

### 5.4. Two-Electrode Voltage Clamp Measurements

Two-electrode voltage clamp experiments were performed three or four days after the microinjection of cRNA, as previously described [66]. Low [K^+^] solution contained (in mM): NaCl 95.4, KCl 2, CaCl_2_ 1.8, HEPES 5 (pH 7.5 adjusted with NaOH). High [K^+^] solution contained 80 mM K^+^ (78 mM Na^+^ of the low [K^+^] solution was replaced with K^+^). For the measurements with Cs^+^ as the charge carrier, the zero [Cs^+^] solution (also called *NMDG solution* or simply *NMDG* in the text) contained (in mM): N-methyl-D-glucamine (NMDG) 97.4, CaCl_2_ 1.8, HEPES 5 (pH 7.5 adjusted with HCl). High [Cs^+^] solution contained 80 mM Cs^+^ (and 17.4 mM NMDG^+^). Background K^+^ and Cs^+^ currents were measured at the end of 300 ms-long voltage steps to −100 mV applied in every 4 s, with the exception of the characterization of voltage-dependence, where the details of the voltage step protocol are given in the figure legend. In some experiments, TMEM175 currents of the same cell were measured twice. The oocyte was removed from the recording chamber between the measurements, and incubated at 18 °C, in 0.1–0.2 mL MB-HK solution, supplemented with the appropriate pharmacological agent as indicated in the *Results* section.

### 5.5. Transfection with TMEM175 cDNA, and Whole Cell Patch Clamp Measurements

HEK293T cells were transfected with 0.5 µg TMEM175-pIRES-CD8 plasmid and 2 µL Invitrogen Lipofectamine 2000 transfection reagent (Thermo Fisher Scientific) per 35 mm petri dish in DMEM without FCS (Dulbecco′s Modified Eagle′s Medium, no Fetal Calf Serum) for 5 h. After the transfection, the cells were incubated in DMEM supplemented with 10% FCS and next day 80 µM Dynasore was added to this medium. Electrophysiology was performed on the second day after transfection. The cells were selected for the measurement with anti-CD8-coated resin (Dynabeads™ CD8 Invitrogen, Thermo Fisher Scientific, #11147D).

Plasma membrane currents of HEK293T cells were recorded with RK-400 amplifier (Biologic, Claix, France) using microelectrodes made of borosilicate glass (World Precision Instruments, BF120-69-10) with a resistance of 3 to 6 MΩ when fire-polished and filled with the pipette solution. The pipette solution contained (in mM): CsCl 140, MgCl_2_ 2, EGTA 5, HEPES 10 (pH 7.3 with NaOH). The bath solutions contained 140 mM NaCl or CsCl in addition to 4 mM MgCl_2_, 1 mM CaCl_2_ and 10 mM HEPES (pH 7.4 with NaOH). The 4-aminopyridine inhibitor (4-AP) was dissolved in the bath solution containing Cs^+^, at a concentration of 1 mM. The experiments were carried out at room temperature, and solutions were applied by gravity-driven perfusion systems. The currents were low-pass filtered at 1 kHz and digitally sampled at 2.5 kHz by Digidata Interface 1440A (Molecular Devices, San Jose, CA, USA). Recording was performed using WinWCP 5.5.4 software (John Dempster, University of Strathclyde, Glasgow, UK) and data were analyzed by pCLAMP Clampfit 11 (Molecular Devices).

### 5.6. Cleavage of Surface Proteins with Proteinase K, and Anti-HA Immunoblot

The TMEM175 construct with intracellular (N-terminal) double HA-tag was expressed in *Xenopus* oocytes. Two days after the injection of cRNA, the cells were treated with 10 μM dyngo-4a or the vehicle DMSO for 20 h. The groups (each containing 19 oocytes) were or were not digested with proteinase K (Thermo Scientific, #EO0491, 120 μg in 400 μL of the low [K^+^] solution described in the “Two-electrode voltage clamp measurements” section) for 45 min at room temperature (22 °C). Subsequently, the groups of oocytes were homogenized in 1 mL of ice-cold lysis solution containing: HEPES (20 mM), imidazole (40 mM), EDTA (10 mM), phenylmethylsulfonyl fluoride (PMSF, 5 mM), benzamidine (1 mM), leupeptine (5 μg/mL), pH 7.9, (adjusted with NaOH), with 20 strokes in a glass Potter homogenizer. The homogenates were centrifuged twice at 1000× *g* for 10 min at 4 °C, in order to remove yolk granules, nuclei and the floating lipid layer. Finally, the supernatant was centrifuged at 16,000× *g* for 10 min at 4 °C, the crude plasma membrane fraction was dissolved in 80 μL of SDS loading buffer containing Tris (0.31 M, pH 6.8, with HCl), SDS (10%), glycerol (50%), mercaptoethanol (25%), and bromphenol blue (0.2%), and stored at −80 °C.

Seven μL denatured samples for each group were separated by SDS-PAGE on 10% gels, and transferred to nitrocellulose membranes (Amersham Protan™ 0.2 μm NC, GE Healthcare Life Sciences). The transfer (tank blotting) was performed overnight at 50 V in Towbin buffer containing 25 mM Tris, 192 mM glycine, pH 8.6 ± 0.2, and 20% methanol. The non-specific binding sites of the membrane were blocked with 0.2 g/10 mL bovine serum albumin, 0.5 g/10 mL non-fat dry milk, and 0.2% Tween-20 in PBS (phosphate-buffered saline). The primary antibody (mouse monoclonal anti-HA IgG1, #26183, Clone 2–2.2.14, diluted 10,000×; Thermo Scientific, RRID:AB_10978021) was applied for 1 h at room temperature in PBS containing 10% blocking buffer. The secondary antibody (goat-anti-mouse IgG (H + L), horseradish peroxidase conjugate, R-05071-500; Advansta, Menlo Park, CA, RRID:AB_10718209) was applied for 1 h under conditions similar to those of the first antibody. The membrane was washed once after the blocking phase and four to six times for 5–10 min in 20–30 mL of PBS containing 0.1% Tween-20 after the antibodies. The bands were visualized by the enhanced chemiluminescence detection method (WesternBright ECL HRP, Advansta) according to the manufacturer’s instructions. Densitometry analysis was performed with ImageJ 1.47v software written by Wayne Rasband (Research Services Branch, NIH, Bethesda, MD, USA). The N-terminally double-HA-tagged TMEM175 protein runs close to the 47 kD marker band, instead of the calculated 58.5 kD molecular weight, probably because of the high (≈48% amino acid sequence) proportion of hydrophobic transmembrane segments in the protein.

### 5.7. Luminometric Detection of Extracellularly HA-Tagged TMEM175 Constructs

Two days after the injection of cRNA, the *Xenopus* oocytes, expressing the TMEM175 constructs with EC HA-tag(s), were treated with 10 μM dyngo-4a or with the vehicle DMSO for 20 h. The cells were fixed for 15 min at room temperature in 4% paraformaldehyde dissolved in XenPBS, containing 100 mM NaCl and 10 mM phosphate, pH 7.4 (NaOH). The non-specific binding sites of the fixed cell surface (9–10 cells in each group) were blocked with 0.2 g/10 mL bovine serum albumin, and 0.5 g/10 mL non-fat dry milk in XenPBS (blocking buffer, 0.5 mL) for 40 min at room temperature. The primary and secondary antibodies were the same as in the anti-HA immunoblot reaction (see in Section 5.6), however, they were diluted 5000× in 0.5 mL XenPBS containing 10% Twin-20-free blocking buffer. The cells were washed three times in 1 mL XenPBS after the fixation, once after the blocking phase, and four times for 5–10 min after the antibodies.

The cells were transferred into a white, flat bottom, 96-well plate and 75 μL ECL reagent was added to each well (WesternBright ECL HRP, Advansta). The luminometric signals were detected with a Varioskan Flash™ microplate reader (Thermo Scientific) five times for 0.5 s/well (one oocyte per well). Care was taken to microinject the animal pole (middle of the brown hemisphere) of the cell with cRNA in these experiments, and to position the oocyte with the animal pole toward the detector of the instrument.

### 5.8. Analysis of HA-Tagged TMEM175 Constructs by Confocal Microscopy

Two days after the injection of HA_2_-TMEM175, HA_2_-CD8-TMEM175, or loopHA-TMEM175 cRNA, the *Xenopus* oocytes were treated with 10 μM dyngo-4a or with the vehicle DMSO for 20 h. The cells were fixed for 20 or 30 min at room temperature in 4% paraformaldehyde dissolved in XenPBS (see in Section 5.7). In the experiment with HA_2_-TMEM175, the plasma membrane of the cells were (or were not) permeabilized with Triton X-100 (0.3%) in XenPBS for 10 min. The non-specific binding sites of the fixed cell surface were blocked with 0.2 g/10 mL bovine serum albumin in XenPBS (blocking buffer, 0.5 mL) for 60 min at room temperature. The primary antibody was the same as in the anti-HA immunoblot reaction (see in Section 5.6), however, it was diluted 1000× in 0.5 mL XenPBS containing 10% (Twin-20-free) blocking buffer. The secondary antibody was F(ab’)2-goat or chicken anti-mouse IgG (H + L) cross-adsorbed secondary antibody, Alexa Fluor 488 (#A-11017 or #A-21200, respectively, Thermo Scientific, RRID:AB_2534084 or RRID:AB_2535786) at 1500× dilution in 0.25 or 0.5 mL XenPBS containing 10% (Twin-20-free) blocking buffer. The cells were washed three times in 1 mL XenPBS after the fixation, once after the permeabilization, once after the blocking phase, and three or four times for 5 min after the antibodies. The cells were examined by a Nikon A1plus, Ti2 Eclipse confocal microscope, by using the NIS-Elements software (5.11.02).

### 5.9. Data and Statistical Analysis

Data are expressed as mean ± S.D. The statistical difference was considered to be significant at *p* < 0.05. Normality of data distribution was estimated by the Shapiro–Wilk test and the homogeneity of variance by Levene test. If the Shapiro–Wilk test showed significance in any groups, then the nonparametric Mann–Whitney U-test was used for two groups or the Kruskal–Wallis ANOVA followed by the multiple comparison of mean ranks for all groups. If Levene test resulted in significance, then the heteroscedastic (Welch-Satterthwaite corrected) *t*-test was used for two groups or Welch’s analysis of variance (ANOVA) followed by Dunnett T3 post hoc test for multiple comparisons. Otherwise, Student’s *t*-test for independent groups, or one-way ANOVA followed by Tukey HSD post hoc test were applied. Post hoc tests were conducted only if ANOVA resulted in significant *p* value. In some cases, paired *t*-test was used, as required. In two experiments, logarithmic transformation was applied to reduce heteroscedasticity and generate a Gaussian-distributed data set amenable to parametric analysis. Statistical calculations were performed in Statistica 13.4 (TIBCO Software, Tulsa, OK, USA), or SPSS Statistics 27.0 (IBM Corporation, Armonk, NY, USA). Non-linear curve fitting of the dose-response curves were performed by least squares method (Origin 6.0; OriginLab Corp., Northampton, MA, USA). We applied the Hill equation of the form α + (β − α)/(1 + (K/c)^n^), where α is the baseline, β is the final level of the effect at high concentration, c is the concentration, K is the concentration at which half maximal effect is evoked, and n is the Hill coefficient.

## Data Availability

Most data is contained within the Results section of this article. Additional data are available on request from the corresponding author.

## Supplementary Materials

The following are available online at https://www.mdpi.com/article/10.3390/ijms221910515/s1.
