# Supplementary material for: Translocation of TMEM175 Lysosomal Potassium Channel to the Plasma Membrane by Dynasore Compounds
Source: Int J Mol Sci. 2021 Sep 29;22(19):10515. doi: 10.3390/ijms221910515 (PMC8508992; doi:10.3390/ijms221910515)
Supplement: Supplementary file 1 [file ijms-22-10515-s001.zip › ijms-1347946-supplementary.pdf]

S1

Dynasore (80 μM)

Cell preparation	Average current (μA)	n (number of measured oocytes)	EC solution (80 mM)	Normalized to K+ current (Cs+ current x 0.45)	
1	0.43	14	K+	0.43	Min.
2	4.60	7	Cs+	2.07	
3	2.19	10	K+	2.19	
4	6.20	7	Cs+	2.79	
5	7.02	3	Cs+	3.16	
6	8.91	5	Cs+	4.01	
7	4.02	23	K+	4.02	
8	9.55	7	Cs+	4.30	
9	5.26	17	K+	5.26	
10	15.50	3	Cs+	6.98	
11	22.48	4	Cs+	10.11	
12	13.11	4	K+	13.11	
13	31.25	4	Cs+	14.06	
14	31.84	5	Cs+	14.33	
15	32.53	4	Cs+	14.64	
16	35.83	5	Cs+	16.12	Max.

Dyngo-4a (10 μM)

Cell preparation	Average current (μA)	n (number of measured oocytes)	EC solution (80 mM)	
1	6.61	11	K+	Min.
2	7.91	10	K+	
3	9.97	5	K+	
4	10.25	6	K+	
5	10.72	7	K+	
6	11.40	4	K+	
7	12.89	4	K+	
8	21.93	8	K+	
9	24.29	7	K+	
10	31.03	7	K+	
11	35.26	4	K+	
12	35.71	7	K+	
13	37.57	5	K+	
14	42.69	5	K+	
15	47.12	10	K+	Max.

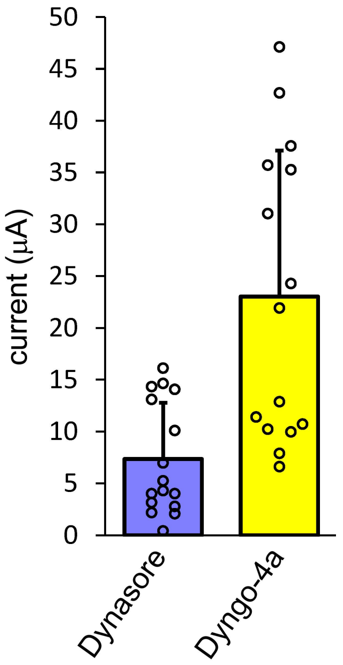

Supplementary figure S1. TMEM175 currents after dynasore or dyngo-4a in different cell preparations. Average TMEM175 currents are compared in the different oocyte preparations after the treatment with dynasore (80 μM, upper table) or dyngo-4a (10 μM, lower table). The currents were measured at -100 mV. Where Cs^{+} was used as the charge carrier, the data are normalized to K^{+} current, considering that TMEM175 potassium current is 45 % of the cesium current under these recording conditions. The data are also shown as a column graph. The difference is statistically significant ($p<0.002$, Mann-Whitney U test). These results confirm the conclusion of Fig. 1.F that dyngo-4a increases TMEM175 current to higher values than dynasore, also at the level of different cell preparations.

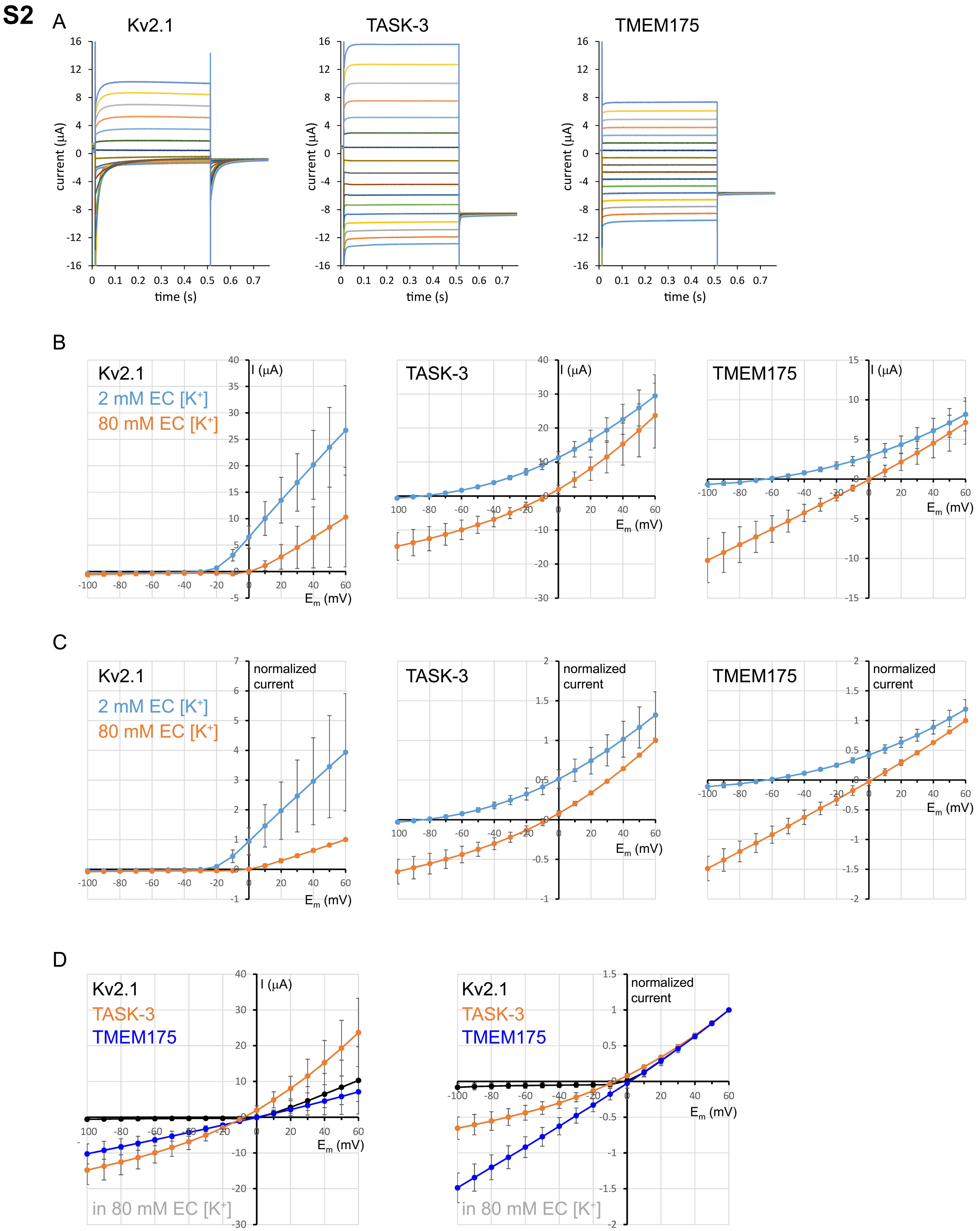

Supplementary figure S2. Comparison of Kv2.1, TASK-3 and TMEM175 K$^+$ currents.

A. The same currents as in Figure 2. A-C are shown with identical y-axis range.

B. Average Kv2.1 (n=5), TASK-3 (n=5), and TMEM175 (n=6) currents were calculated under identical conditions in three groups of oocytes from the same cell preparation. The current-voltage (I-V) relationships were determined in 2 and 80 mM EC [K$^+$] for each channel type. TMEM175 currents were induced by dyngo-4a (10 µM, 20 h).

C. The curves were normalized to the value measured at +60 mV in 80 mM EC [K$^+$]. Note that a major part of the standard deviation (S.D.) in panel B was caused by the different expression levels in the oocytes.

D. The current and normalized current data for the three channel types in 80 mM EC [K$^+$] are shown in the same graphs, respectively. The curves are repeated from panels B and C. The normalized currents are significantly different at -100 mV ($p<0.05$, Mann-Whitney U test, after Bonferroni correction).

S3

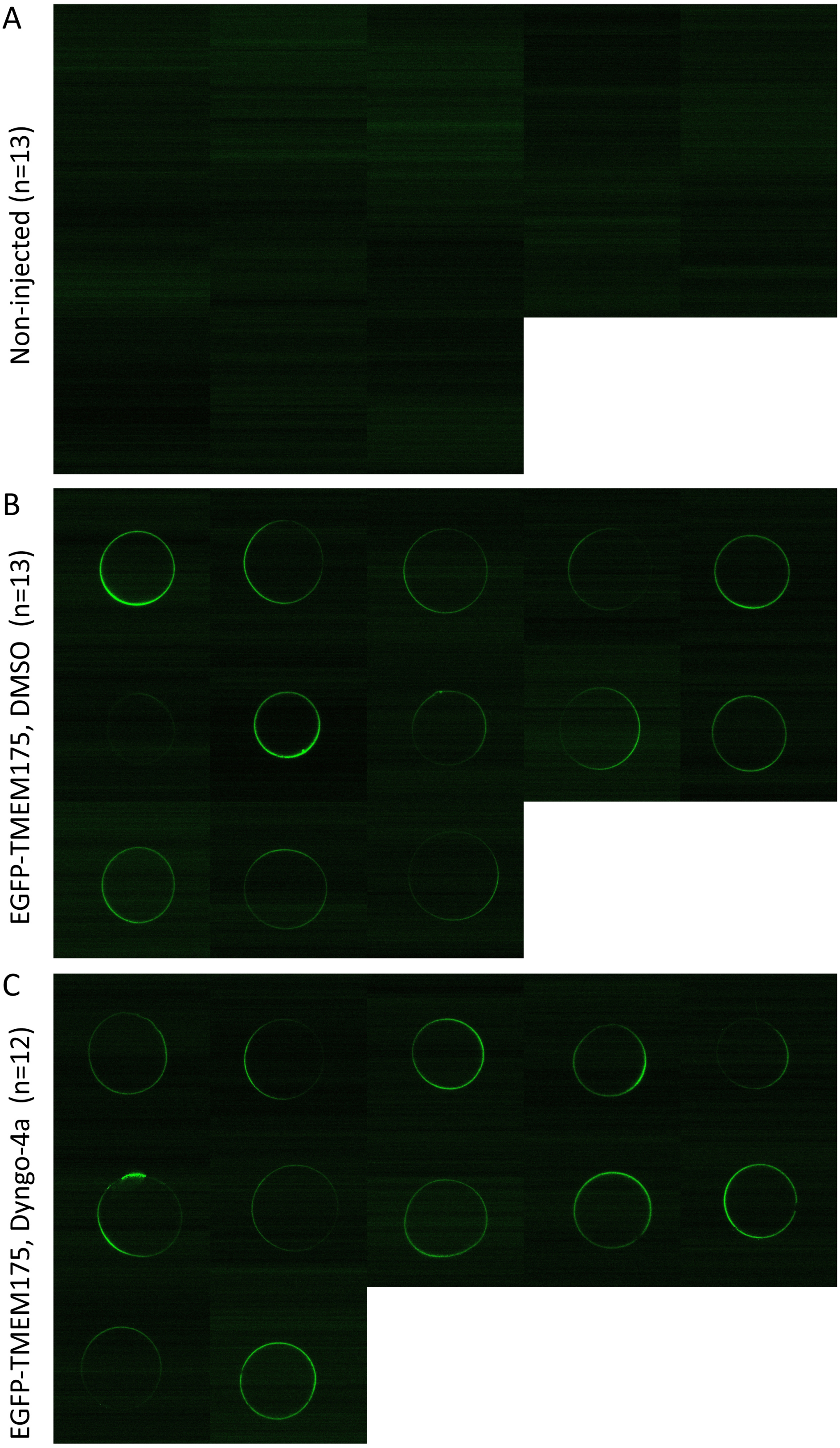

Supplementary figure S3. The detection of EGFP-TMEM175 fluorescence by confocal microscopy is not an adequate method to estimate the surface expression of the channel in *Xenopus* oocytes.

Mouse TMEM175 N-terminally fused to enhanced green fluorescent protein (EGFP) was expressed in *Xenopus* oocytes, and the cells were treated with Dyngo-4a (10 µM, 20 h, panel C), or with the vehicle DMSO (panel B). The fluorescence of non-injected cells was measured under identical conditions with confocal microscopy (as a control, panel A). The fluorescence intensity of both the DMSO- and Dyngo-treated cells was significantly higher than that of the non-injected oocytes ($p<0.0002$, multiple comparisons after Kruskal-Wallis ANOVA). Thus, EGFP-TMEM175 was expressed in this experiment. We also verified that EGFP-TMEM175 induced K^+ currents in the plasma membrane only after the application of Dyngo-4a (data not shown). However, the fluorescence was not different between the DMSO and Dyngo groups (we do not show the details of the analysis). The most probable explanation for the lack of difference is that the detection of GFP signal does not distinguish between the channels located in the plasma membrane or in the intracellular membranes in the proximity of the plasma membrane. We used the lowest possible laser intensity for the detection of GFP fluorescence. Living cells were measured.

S4

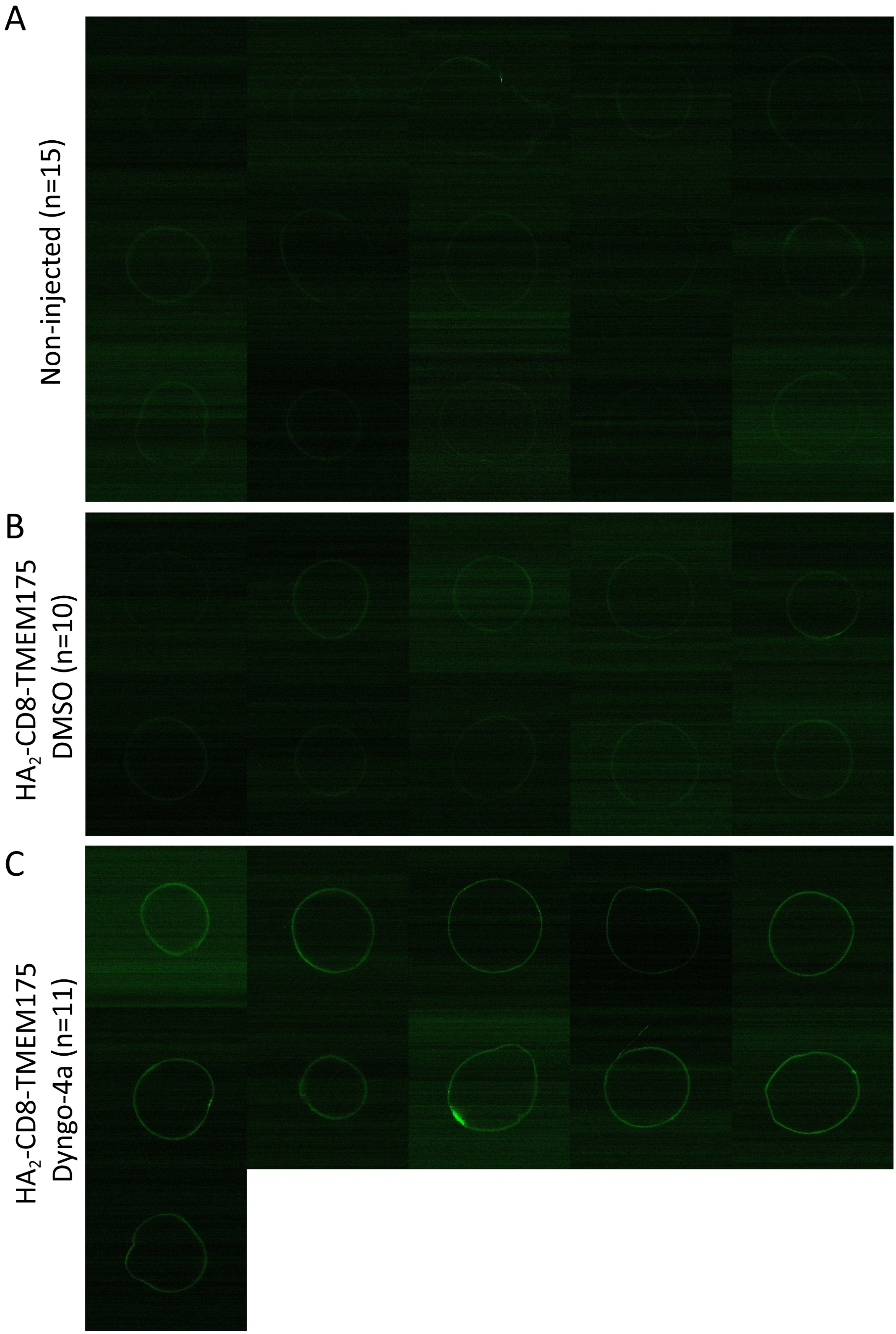
A
Non-injected (n=15)
B
HA_2-CD8-TMEM175
DMSO (n=10)
C
HA_2-CD8-TMEM175
Dyngo-4a (n=11)

S4

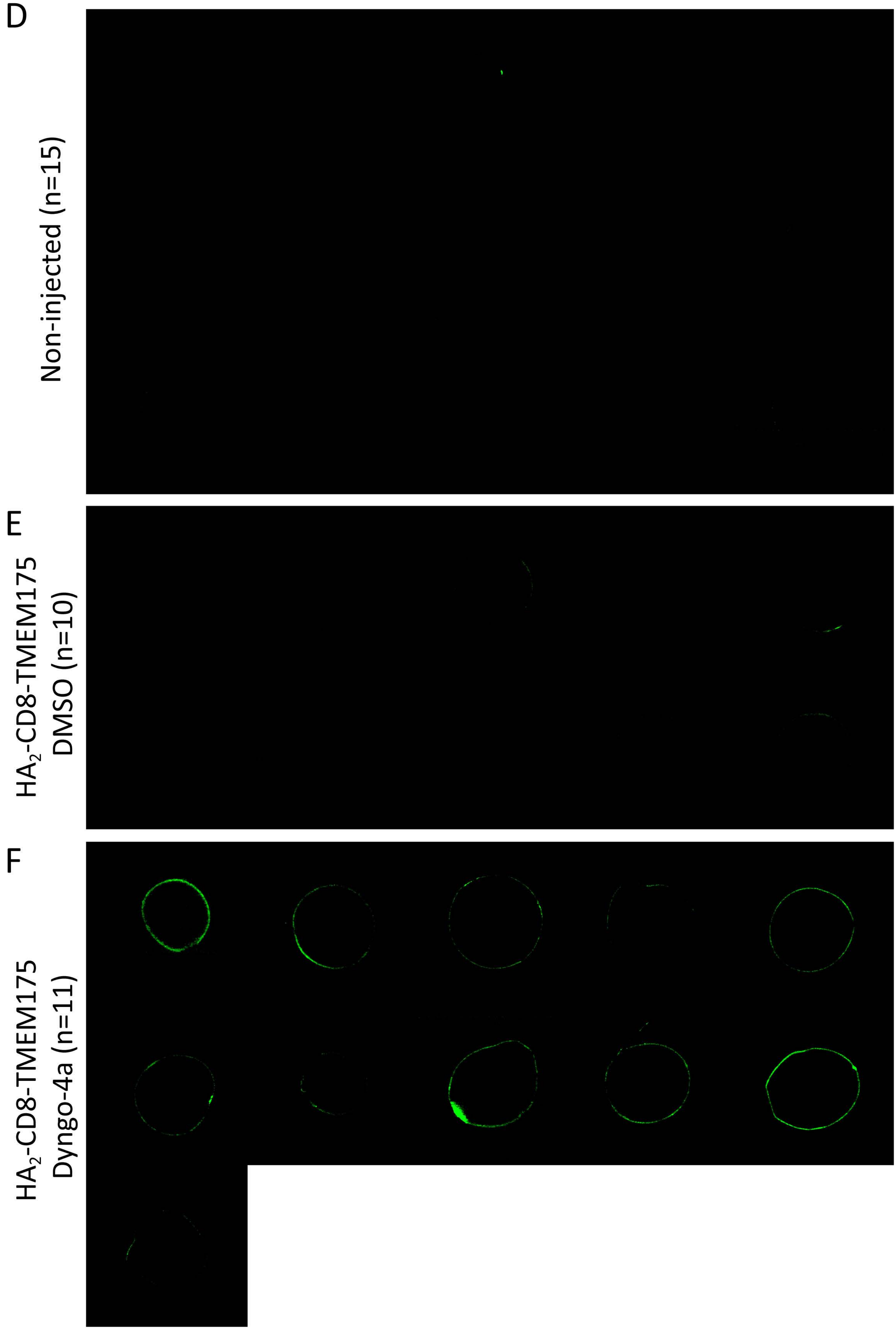
D
Non-injected (n=15)
E
HA$_2$-CD8-TMEM175
DMSO (n=10)
F
HA$_2$-CD8-TMEM175
Dyngo-4a (n=11)

Supplementary figure S4.

Dyngo-4a increases the abundance of the HA_2-CD8-TMEM175 construct on the cell surface.

The extracellularly HA-tagged HA_2-CD8-TMEM175 construct (see Figure 4.E in the main text) was detected by anti-HA immunofluorescence and confocal microscopy imaging. The *Xenopus* oocytes were fixed, but not permeabilized, in order to prevent the access of the antibodies to intracellular channels.

A. Under these conditions, the non-injected oocytes produced faint fluorescence on their surface after the immunostaining. This signal was in the range of the background noise of the confocal microscope detector system.

B. The oocytes expressing HA_2-CD8-TMEM175, and treated with the vehicle DMSO overnight, produced similarly low signals as the control non-injected cells.

C. The oocytes expressing HA_2-CD8-TMEM175, and treated with Dyngo-4a (10 μM, 20 h), were characterized by brighter fluorescence on their circumference than the control non-injected or DMSO-treated cells.

D.-F. Since the fluorescence intensity of the pixels at cell surface was identical to the intensity of the brightest points of the background, it was challenging to extract useful quantitative information from the images shown in panel A-C. We used spatial filtering (the Smooth function of ImageJ software five times) in order to suppress the solitary high intensity pixels of the background, while maintaining high intensity adjacent pixels at the cell surface. This allowed lower cutoff levels for the filtering of the background noise. In panels D-F, we show the result of this image processing of panels A-C, respectively. The cumulative fluorescence intensity was calculated from the processed images, and used for the statistical analysis shown in Figure 5 in the main text.

S5

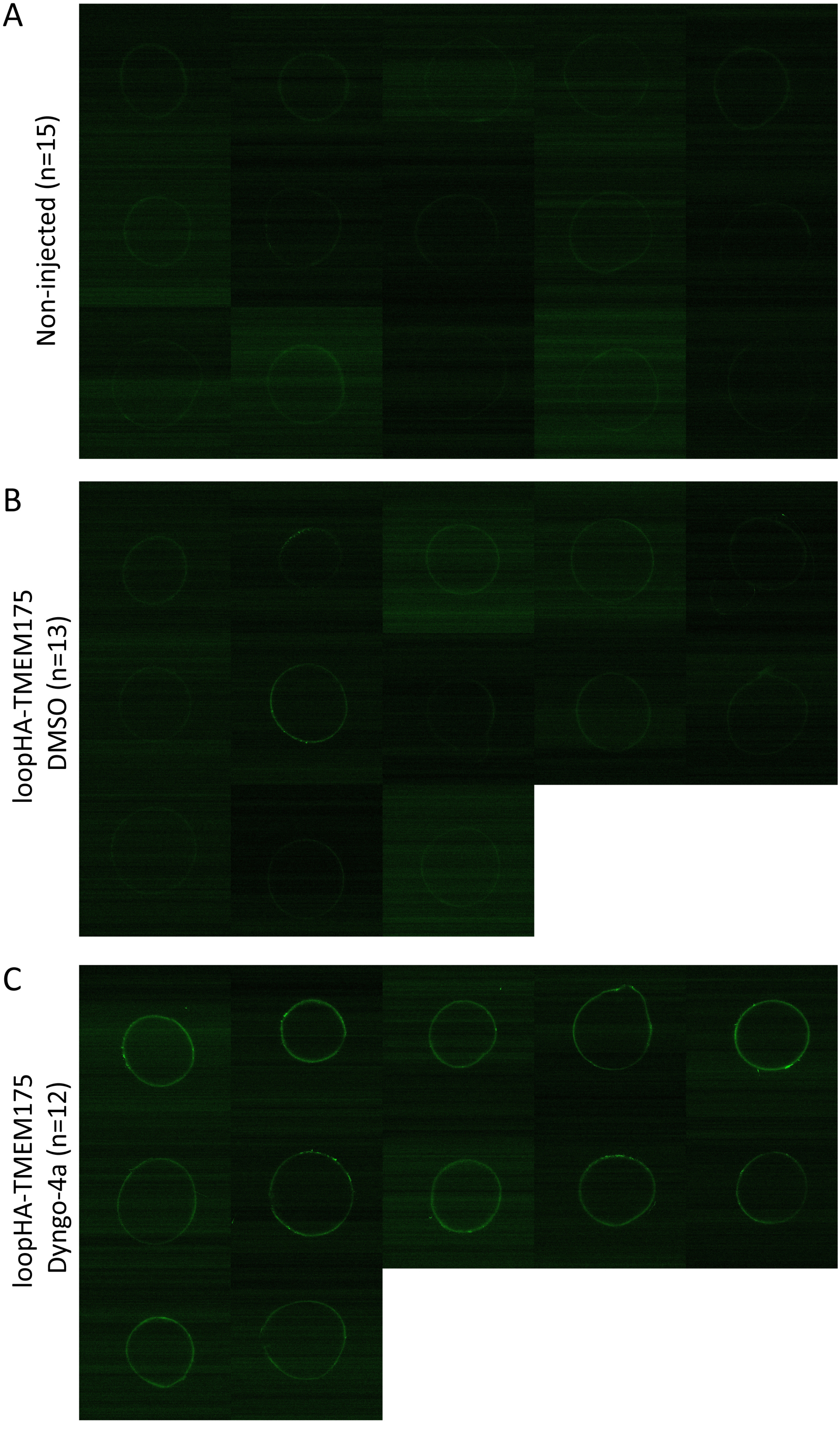
A
Non-injected (n=15)
B
loopHA-TMEM175
DMSO (n=13)
C
loopHA-TMEM175
Dyngo-4a (n=12)

S5

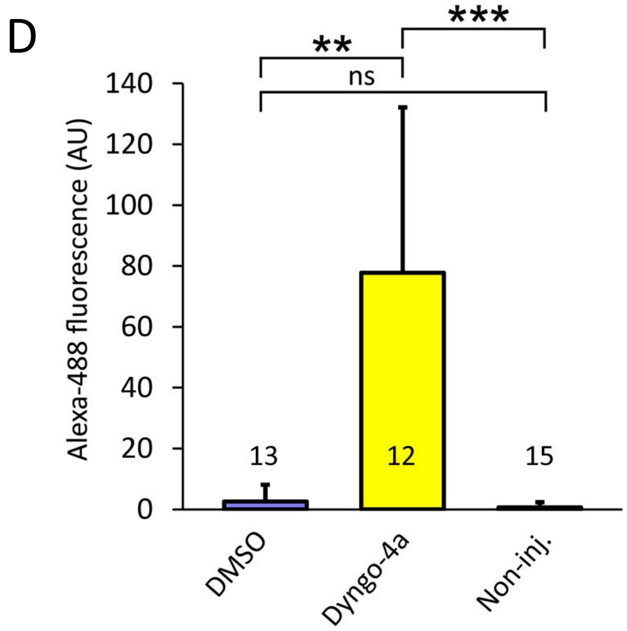

Supplementary figure S5.

Dyngo-4a increases the abundance of the loopHA-TMEM175 construct on the cell surface.

A similar experiment as documented in Figure 5 (in the main text) and supplementary figure S4 was performed with the loopHA-TMEM175 construct (see Figure 4.H in the main text). The loopHA-TMEM175 is also extracellularly HA-tagged, accordingly, the cells were fixed, but not permeabilized. The cells expressing loopHA-TMEM175 and treated with Dyngo-4a (10 µM, 20 h) clearly show higher fluorescence intensity (panel C) than the non-injected control cells (panel A), or the cells expressing loopHA-TMEM175 and treated with DMSO (panel B). These data confirm the results obtained with the structurally different HA_2-CD8-TMEM175. Dyngo-4a increases the number of TMEM175 channels in the plasma membrane irrespective of the location of the HA tag introduced into the construct. The numbers above (or within) the columns indicate sample size (panel D). **$p<0.001$, ***$p<10^{-4}$, multiple comparison after Kruskal-Wallis ANOVA, ns: not significant.

S6

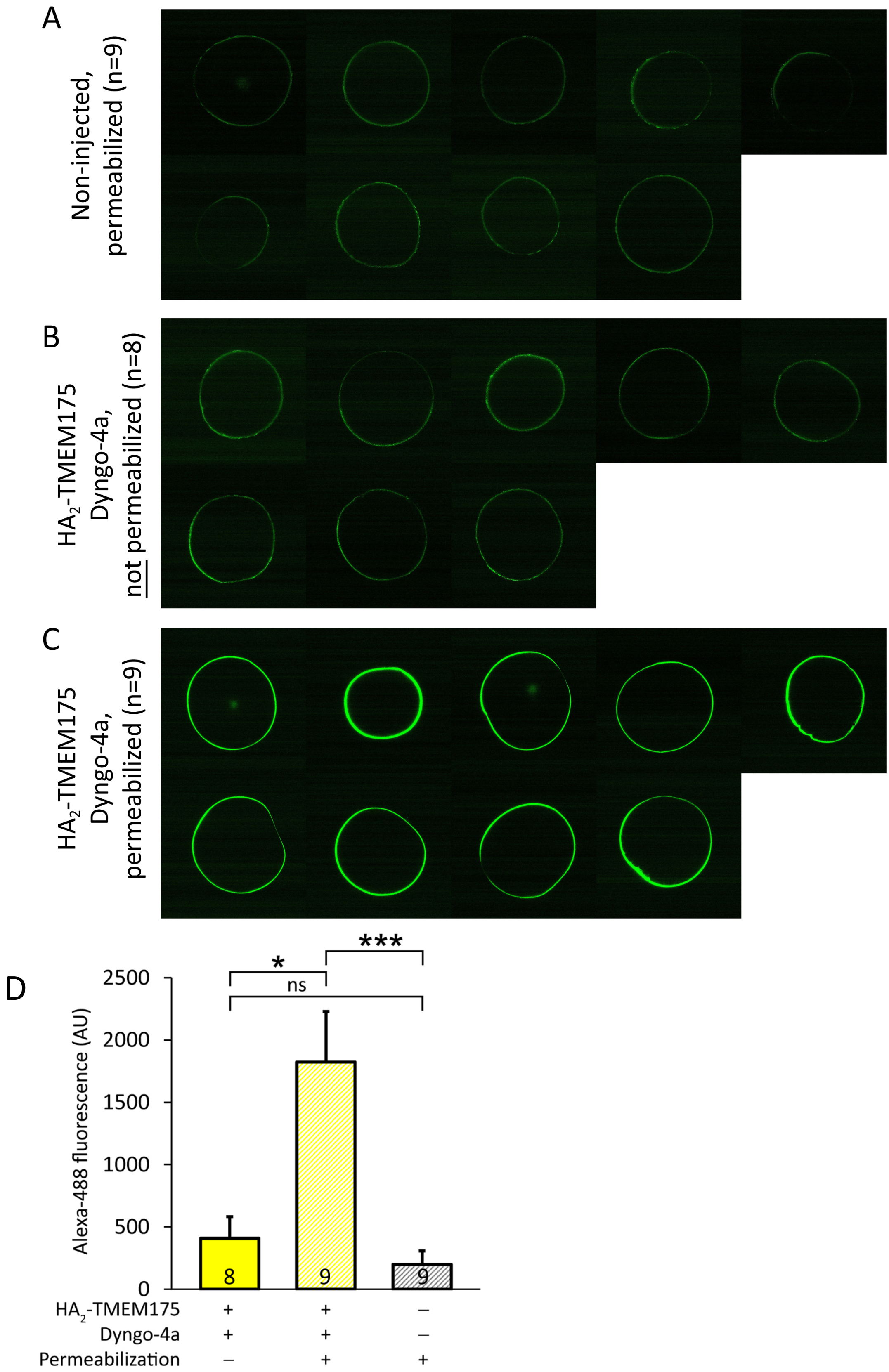

Supplementary figure S6.

The HA-tag of the HA_2-TMEM175 construct is accessible after the permeabilization of the plasma membrane. The HA_2-TMEM175 construct contains intracellular HA-tags (see Figure 4.A in the main text). Accordingly, the *Xenopus* oocytes expressing this construct showed intense fluorescence after the permeabilization of the plasma membrane with 0.3% Triton X-100 for 10 min (panel C, the cells were pretreated with Dyngo-4a, 10 µM, 20 h). The cells expressing HA_2-TMEM175, and pretreated with Dyngo-4a, showed much weaker signal without (panel B) than with permeabilization (panel C). The non-injected control cells also showed weak signals after permeabilization (panel A), indicating that the expression of the HA_2-TMEM175 construct was required for the intense fluorescence. The robust signal of HA_2-TMEM175 after permeabilization (panel C) may also correspond to channels located in the intracellular membranes. The numbers in the columns indicate sample size (panel D). *p<0.02, ***p<10-4, multiple comparisons after Kruskal-Wallis ANOVA, ns: not significant.

S7

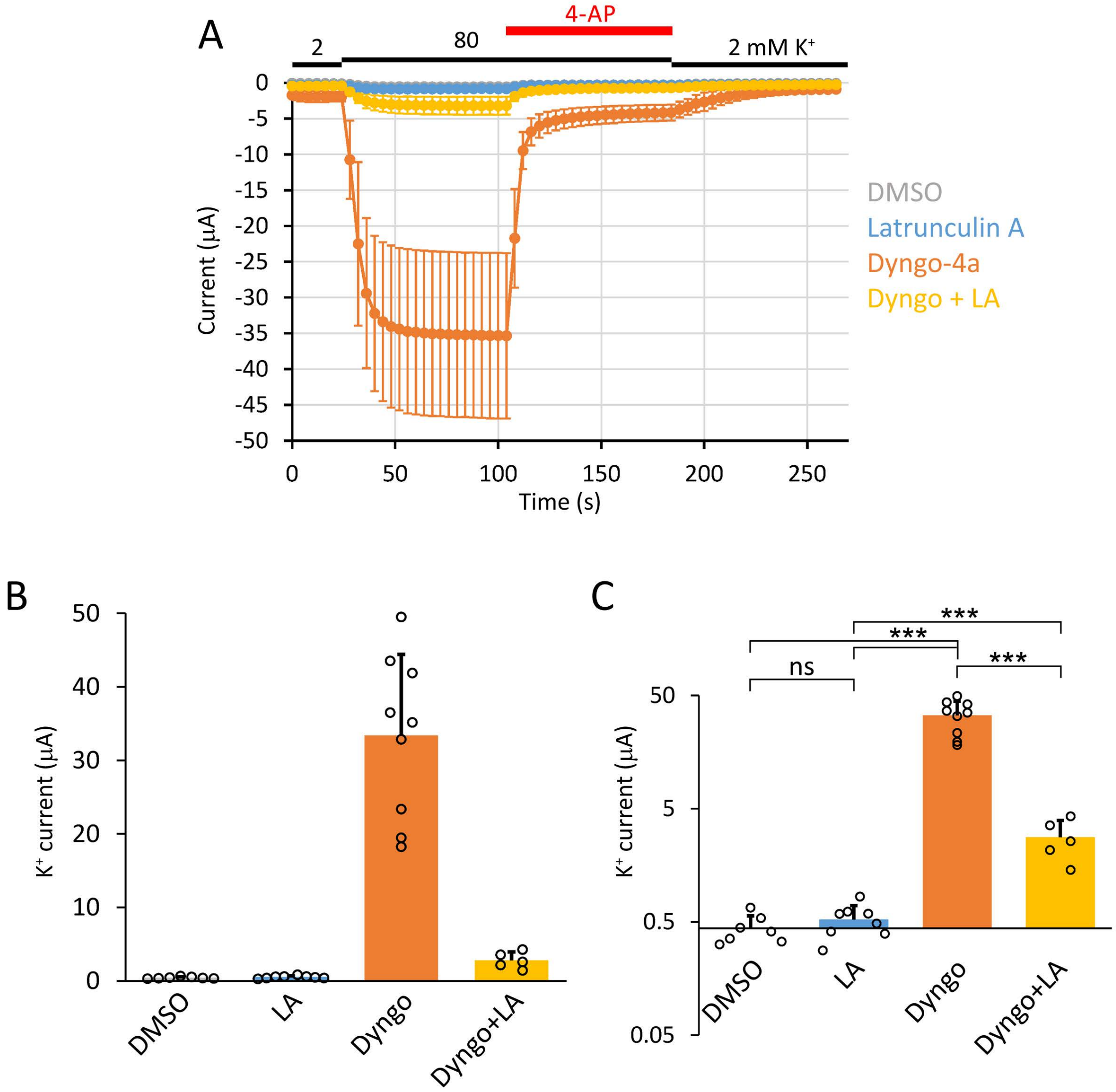

Supplementary figure S7.

Latrunculin A does not induce TMEM175 current in the plasma membrane.

A. Four groups of oocytes expressing mouse TMEM175 were treated with *DMSO* (negative control), Latrunculin A (*LA*, 2 µg/ml), Dyngo-4a (*Dyngo*, 10 µM, positive control), or Dyngo-4a plus Latrunculin A (*Dyngo+LA*) for 20 h. After the pretreatment, TMEM175 currents were measured at -100 mV by changing the extracellular K^+ concentration to 80 mM from 2mM, as indicated above the *graph*. The specificity of the current was further verified by the application of 4-aminopyridine (4-AP, 1 mM, *red bar*). The robust inhibition of the actin cytoskeleton with Latrunculin A did not induce TMEM175 current in the plasma membrane, arguing against the hypothesis that the Dynasore compounds induce TMEM175 current via their off-target effect on the actin cytoskeleton. In contrast, Latrunculin A significantly inhibited the effect of Dyngo-4a, suggesting that the actin cytoskeleton is required for the expression or translocation of the channel into the plasma membrane.

B. TMEM175 K^+ currents in the measured cells, and the average currents in the four groups are plotted, using a linear vertical scale.

C. The same data as in panel B are plotted on a logarithmic scale to clearly visualize the differences among the groups. Statistical analysis was performed on log transformed data. ***p<0.0002, ANOVA, Tukey HSD post hoc test, ns: not significant.

Table 1. Potassium currents of *Xenopus* oocytes expressing TMEM175 were measured after the pretreatment with the different combinations of the dynamin-inhibitor Dyngo-4a (10 μM), the protein kinase B (PKB) activator SC79 (20 μM), and allosteric PKB inhibitor MK2206 (20 μM) for 20 h, as indicated in the table. The data correspond to Figure 8.B and C column graphs in the main text.

Group	Dyngo-4a	SC79	MK2206	K^+ current (μA) average ± S.D.	Number of cells
1.	-	-	-	0.20 ± 0.04	6
2.	-	+	-	1.13 ± 0.85	6
3.	-	+	+	0.81 ± 0.91	7
4.	+	-	-	31.03 ± 26.74	7
5.	+	-	+	6.54 ± 1.25	7
6.	+	+	-	12.97 ± 7.83	7

Table 2. Potassium currents of *Xenopus* oocytes expressing only TMEM175, or coexpressing a constitutively active, deleted version of protein kinase B (ΔPKB) with the channel were measured after the pretreatment with the different combinations of Dyngo-4a (10 μM), and MK2206 (20 μM) for 20 h, as indicated in the table. The data correspond to Figure 9.B and C column graphs in the main text.

Group	ΔPKB	Dyngo-4a	MK2206	K^+ current (μA) average ± S.D.	Number of cells
1.	-	-	-	0.10 ± 0.06	5
2.	-	+	-	21.93 ± 12.25	7
3.	-	+	+	4.33 ± 2.88	8
4.	+	-	-	1.27 ± 0.75	5
5.	+	+	-	31.73 ± 17.90	9
6.	+	+	+	13.44 ± 7.00	8
